# Multicenter analysis of neutrophil extracellular trap dysregulation in adult and pediatric COVID-19

**DOI:** 10.1172/jci.insight.160332

**Published:** 2022-08-22

**Authors:** Carmelo Carmona-Rivera, Yu Zhang, Kerry Dobbs, Tovah E. Markowitz, Clifton L. Dalgard, Andrew J. Oler, Dillon R. Claybaugh, Deborah Draper, Meng Truong, Ottavia M. Delmonte, Francesco Licciardi, Ugo Ramenghi, Nicoletta Crescenzio, Luisa Imberti, Alessandra Sottini, Virginia Quaresima, Chiara Fiorini, Valentina Discepolo, Andrea Lo Vecchio, Alfredo Guarino, Luca Pierri, Andrea Catzola, Andrea Biondi, Paolo Bonfanti, Maria C. Poli Harlowe, Yasmin Espinosa, Camila Astudillo, Emma Rey-Jurado, Cecilia Vial, Javiera de la Cruz, Ricardo Gonzalez, Cecilia Pinera, Jacqueline W. Mays, Ashley Ng, Andrew Platt, NIH COVID Autopsy Consortium, COVID STORM Clinicians, Beth Drolet, John Moon, Edward W. Cowen, Heather Kenney, Sarah E. Weber, Riccardo Castagnoli, Mary Magliocco, Michael A. Stack, Gina Montealegre, Karyl Barron, Danielle L. Fink, Douglas B. Kuhns, Stephen M. Hewitt, Lisa M. Arkin, Daniel S. Chertow, Helen C. Su, Luigi D. Notarangelo, Mariana J. Kaplan

**Affiliations:** 1 Systemic Autoimmunity Branch, National Institute of Arthritis and Musculoskeletal and Skin Diseases (NIAMS);; 2 Human Immunological Diseases Section, Laboratory of Clinical Immunology and Microbiology (LCIM), National Institute of Allergy and Infectious Diseases (NIAID); and; 3 LCIM, NIAID, NIH, Bethesda, Maryland, USA.; 4 Axle Informatics, Bethesda, Maryland, USA.; 5 Department of Anatomy, Physiology & Genetics, School of Medicine, and the American Genome Center, Uniformed Services University of the Health Sciences (USUHS), Bethesda, Maryland, USA.; 6 Bioinformatics and Computational Biosciences Branch, Office of Cyber Infrastructure and Computational Biology, NIAID, NIH, Bethesda, Maryland, USA.; 7 Department of Public Health and Pediatric Sciences and; 8 Pediatric Hematology, “Regina Margherita” Children Hospital, University of Turin, Turin, Italy.; 9 Centro di Ricerca Emato-oncologica AIL, Diagnostic Department, ASST Spedali Civili di Brescia, Brescia, Italy.; 10 Department of Translational Medical Sciences, Pediatric Section, University of Naples Federico II, Naples, Italy.; 11 Department of Pediatrics, University of Milano-Bicocca, European Reference Network (ERN) PaedCan, EuroBloodNet, MetabERN, Fondazione MBBM/Ospedale San Gerardo, Monza, Italy.; 12 Department of Infectious Diseases, San Gerardo Hospital–University of Milano-Bicocca, Monza, Italy.; 13 Programa de Inmunogenética e Inmunología Traslacional, Instituto de Ciencias e Innovación en Medicina, Facultad de Medicina Clínica Alemana, Universidad del Desarrollo, Santiago, Chile.; 14 Hospital Roberto del Rio, Santiago, Chile.; 15 Facultad de Medicina Clínica Alemana Universidad del Desarrollo, Programa Hantavirus, Instituto de Ciencias e Innovación en Medicina, Santiago, Chile.; 16 Pediatric Intensive Care Unit, Hospital Exequiel Gonzalez Cortés, Santiago, Chile.; 17 Infectious Diseases Unit, Hospital Dr. Exequiel González Cortés, Región Metropolitana, Chile.; 18 Faculty of Medicine, Universidad de Chile, Santiago, Chile.; 19 National Institute of Dental and Craniofacial Research (NIDCR), NIH, Bethesda, Maryland, USA.; 20 Department of Dermatology, University of Wisconsin School of Medicine and Public Health, Madison, Wisconsin, USA.; 21 Emerging Pathogens Section, Critical Care Medicine Department, Clinical Center, and Laboratory of Immunoregulation, NIAID, NIH, Bethesda, Maryland, USA.; 22 The NIH COVID Autopsy Consortium is detailed in Supplemental Acknowledgments.; 23 The COVID STORM Clinicians are detailed in Supplemental Acknowledgments.; 24 Dermatology Branch, NIAMS;; 25 Molecular Development of the Immune System Section, Laboratory of Immune System Biology, NIAID; and; 26 Division of Clinical Research, NIAID, NIH, Bethesda, Maryland, USA.; 27 Applied/Developmental Research Directorate, Frederick and National Laboratory for Cancer Research, National Cancer Institute (NCI), NIH, Frederick, Maryland, USA.; 28 Laboratory of Pathology, Center for Cancer Research, NCI, NIH, Bethesda, Maryland, USA.

**Keywords:** Infectious disease, Inflammation, Neutrophils

## Abstract

Dysregulation in neutrophil extracellular trap (NET) formation and degradation may play a role in the pathogenesis and severity of COVID-19; however, its role in the pediatric manifestations of this disease, including multisystem inflammatory syndrome in children (MIS-C) and chilblain-like lesions (CLLs), otherwise known as “COVID toes,” remains unclear. Studying multinational cohorts, we found that, in CLLs, NETs were significantly increased in serum and skin. There was geographic variability in the prevalence of increased NETs in MIS-C, in association with disease severity. MIS-C and CLL serum samples displayed decreased NET degradation ability, in association with C1q and G-actin or anti-NET antibodies, respectively, but not with genetic variants of DNases. In adult COVID-19, persistent elevations in NETs after disease diagnosis were detected but did not occur in asymptomatic infection. COVID-19–affected adults displayed significant prevalence of impaired NET degradation, in association with anti-DNase1L3, G-actin, and specific disease manifestations, but not with genetic variants of DNases. NETs were detected in many organs of adult patients who died from COVID-19 complications. Infection with the Omicron variant was associated with decreased NET levels when compared with other SARS-CoV-2 strains. These data support a role for NETs in the pathogenesis and severity of COVID-19 in pediatric and adult patients.

## Introduction

Coronavirus disease 2019 (COVID-19) is caused by infection with severe acute respiratory syndrome coronavirus 2 (SARS-CoV-2) (1, 2). SARS-CoV-2 infection causes mild to severe illness that may progress to acute respiratory distress syndrome (ARDS), multiorgan failure, and death. In severe cases, infection of pneumocytes and associated inflammation result in lung injury, thrombotic microangiopathy, and hypoxemia (3–9). More than 5 million COVID-19 deaths have been reported globally, predominantly among unvaccinated adults with preexisting medical conditions. Although acute SARS-CoV-2 infection is less severe among children (10–12), multi-system inflammatory syndrome in children (MIS-C) (13, 14), characterized by fever, systemic inflammation, and multiorgan dysfunction following COVID-19, may occur (15, 16). Cutaneous manifestations, such as chilblain-like lesions (CLLs) or “COVID toes,” affect primarily children and young adults and are characterized by edema, erythema, violaceous discoloration on the fingers and toes and occasional vesiculation (17–19). The occurrence of CLLs rapidly increased during the COVID-19 pandemic and correlates with confirmed COVID-19 incidence (19). Biopsy of affected tissue may confirm the diagnosis, with superficial and deep dermal lymphocytic infiltrates surrounding eccrine glands and blood vessels (20–23).

CLLs have been observed in patients with mild and asymptomatic COVID-19, and post-mRNA SARS-CoV-2 vaccination, but remain controversial as many patients are PCR negative without seroconversion (24). Chilblains may occur in type I interferonopathies, and affected patients with CLLs can produce increased IFN-α, supporting that CLLs may be associated with rapid clearance of SARS-CoV-2 infection (25).

Increased numbers of activated neutrophils have been described in severe COVID-19 and in MIS-C (26, 27). Emerging evidence suggests that excessive neutrophil extracellular trap (NET) formation plays a key role in COVID-19 pathogenesis (28–30). NETs are extruded by neutrophils as a meshwork of chromatin bound to granule proteins and are synthesized during various infectious and sterile inflammatory conditions (31). Infection with SARS-CoV-2 can trigger NET formation (32). In other conditions, excessive NET formation may cause vascular injury by activating endothelial-mesenchymal transition, triggering death of endothelial cells and vascular smooth muscle cells and promoting coagulation (33–37). NET complexes have been detected in the circulation of adult patients with severe COVID-19 (38), and widespread occlusion of small vessels by aggregated NETs has been visualized in their lungs and kidneys (39, 40). The mechanisms by which NET remnants are elevated in circulation and tissues in COVID-19 require further characterization. In particular, the relative contribution of enhanced NET formation versus impaired NET degradation in COVID-19 remains a matter of debate. Whether NETs are present in asymptomatic patients infected with the virus and the natural history of elevated NET complexes after the virus is cleared and infection resolves also remain unclear. Furthermore, whether patients infected with different variants of the virus differ in their ability to form NETs remains to be determined.

While various studies have been published on adult cohorts, the presence of NETs and the clearance mechanisms of NETs have not been systematically explored in pediatric patients displaying MIS-C or CLLs. Therefore, we analyzed whether children affected with MIS-C or CLLs exhibit evidence of NET dysregulation, compared with adult patients hospitalized with COVID-19, using a multi-cohort approach. Additionally, we evaluated putative mechanisms implicated in NET degradation abnormalities in subsets of patients with COVID-19. Here, we report that levels of NET remnants are elevated in MIS-C and CLLs, in a geographically dependent manner, and in association with specific disease manifestations. Pediatric and adult symptomatic COVID-19 patients displayed impaired NET degradation that did not appear to be genetically driven and was multifactorial. Furthermore, differences in NET levels were observed when comparing different strains of the virus that have been associated with different levels of disease severity and complications. These results highlight a putative pathogenic role of NETs and impairments in NET degradation in pediatric and adult patients affected by COVID-19.

## Results

### NET remnants are elevated in circulation in children with MIS-C and CLLs.

**Figure 1:**
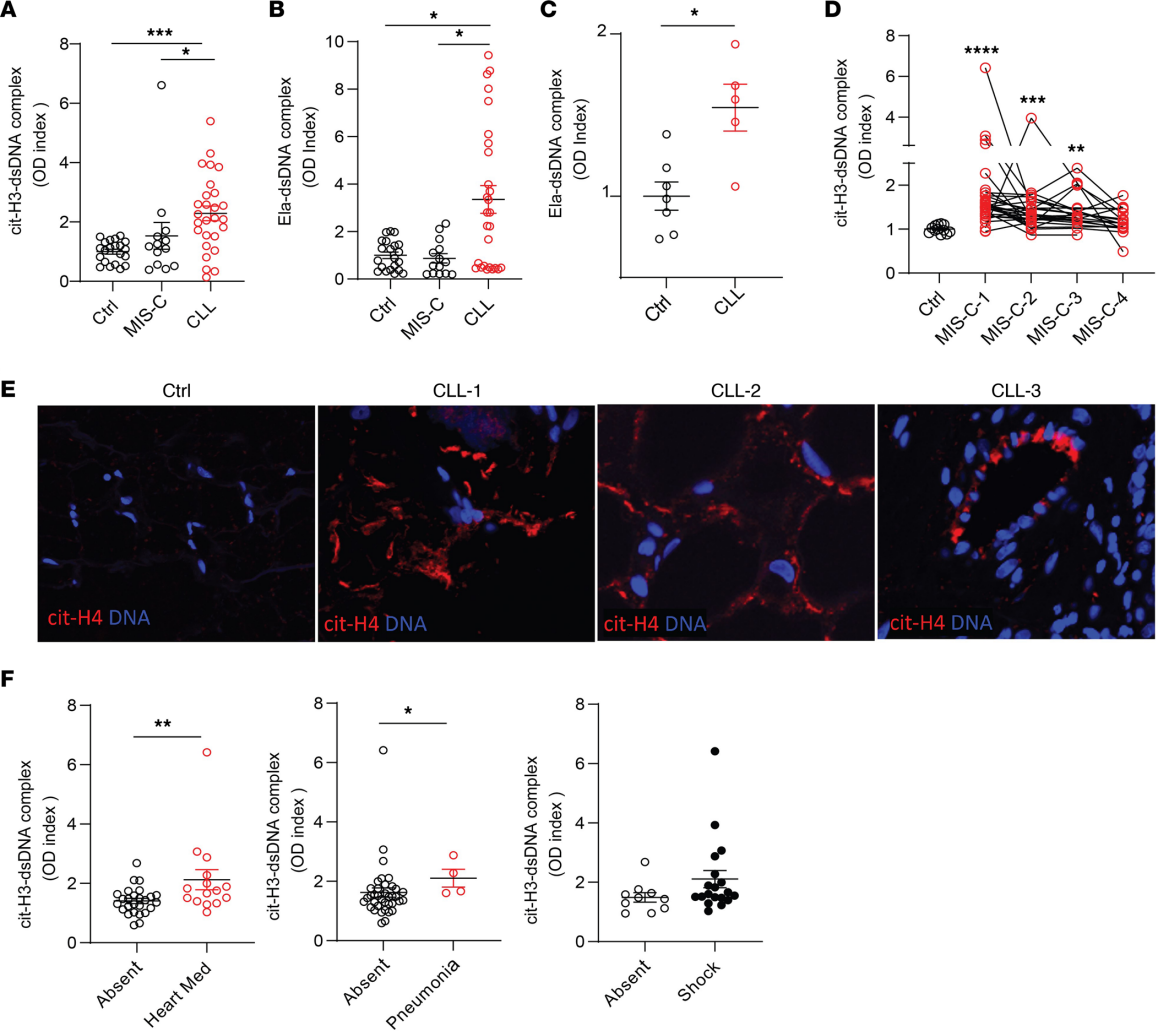
NETs are identified in MIS-C and CLLs. Citrullinated histone H3– and elastase-DNA (citH3-DNA and Ela-DNA) complexes were quantified in serum or plasma MIS-C and CLL samples obtained at 2 different visits for some patients from (**A** and **B**) Italy (MIS-C *n* = 14, CLL *n* = 27, ctrl *n* = 21), (**C**) USA (CLL *n* = 5, ctrl *n* = 5), and (**D**) Chile (MIS-C *n* = 27, ctrl *n* = 12). Mann-Whitney and Kruskal-Wallis analyses were performed. (**E**) Detection of citrullinated histone H4 (citH4, shown in red) and DNA (shown in blue) was performed in skin tissue. Microphotographs depict representative images in 3 CLL and 1 control (from a total of 8 CLL and 2 control specimens). Original magnification, 40×. (**F**) Levels of citH3-DNA complexes were correlated with the absence or presence of inotropes (Heart Med), pneumonia, or shock in Chilean patients with MIS-C. Results are the mean ± SEM. Mann-Whitney analysis was performed. *P < 0.05, **P < 0.01, ***P < 0.001, ****P < 0.0001. Ctrl, controls; OD, optical density.

We quantified NET remnants, as assessed by citrullinated histone H3–DNA (citH3-DNA) and elastase-DNA complexes in pediatric serum or plasma samples collected in medical centers from different regions in Italy, as well as from Chile and the United States. The characteristics of these cohorts are displayed in Table 1. All patients diagnosed with CLLs in Italy and the United States were negative for SARS-CoV-2 by PCR and/or had negative serology, and, to date, none of the patients included in the study have developed evidence of chronic systemic autoimmunity after MIS-C or after CLLs. In the samples from Italy, levels of citH3-DNA and elastase-DNA complexes were significantly higher in children affected by CLLs when compared with healthy controls or patients with MIS-C (Figure 1, A and B). Similarly, samples obtained from children affected by CLLs in the United States displayed significantly elevated levels of elastase-DNA complexes when compared with healthy controls (Figure 1C). While MIS-C samples obtained from Italian cohorts did not show evidence of elevated NET remnants when compared with healthy controls (Figure 1, A and B), a longitudinal assessment of samples obtained from children hospitalized with MIS-C in Chile showed that citH3-DNA complexes were significantly elevated when compared with healthy controls (Figure 1D).

Given that levels of NETs were elevated in circulation in children presenting with CLLs, we evaluated if NETs were present in the affected tissues from biopsies obtained in the United States. All samples had histopathologic features of CLL and were collected after the beginning of the COVID-19 pandemic in 2020. For control skin samples, we used biopsies obtained prior to the onset of the COVID-19 pandemic. As a marker of NETs, skin specimens from CCL patients in the United States were stained for citrullinated histone H4 (citH4), as previously described (41). We detected evidence of NETs infiltrating the CLL tissues but not the control skin (Figure 1E). CitH4-positive structures tended to localize around blood vessels and in the subcutaneous tissue (Figure 1E).

Levels of citH3-DNA complexes in the Chilean MIS-C cohort associated with requiring cardiac inotropes and with development of lung complications (Figure 1F). Furthermore, levels of citH3-DNA complexes displayed a trend to be elevated in those MIS-C patients who developed shock (as assessed by clinical evidence of insufficient tissue perfusion and signs of compensatory mechanisms; Figure 1F). These results suggest that NETs are present in pediatric COVID-19 patients with specific disease manifestations and are associated with disease severity. The results also illustrate geographic variation among pediatric cohorts in their associations between disease manifestations and the detection of NETs in circulation.

### NET degradation is impaired in children with MIS-C and CLLs.

**Figure 2:**
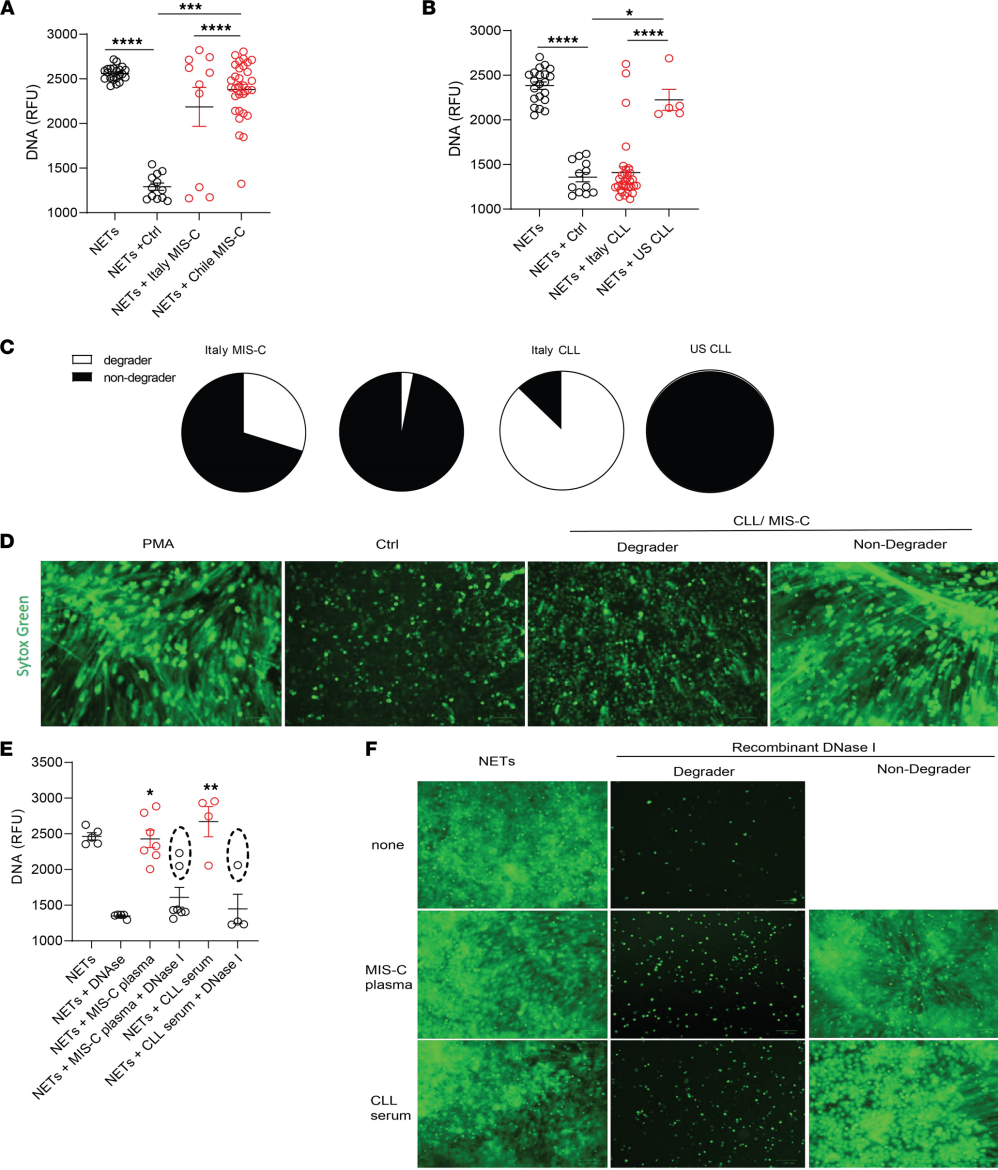
Impaired NET degradation in MIS-C and CLLs. NET degradation capabilities were measured in MIS-C serum or plasma obtained from (**A**) Italy (MIS-C *n* = 12, control *n* = 12) and Chile (MIS-C *n* = 27) and CLLs obtained from (**B**) Italy (CLL *n* = 27, control *n* = 12) and the United States (*n* = 5). Results are the mean ± SEM. Kruskal-Wallis analysis was performed. (**C**) Pie charts representing the proportion of degrader (white) and nondegrader (black) per cohort. (**D**) Representative images of PMA-generated NETs by healthy control neutrophils incubated with serum or plasma from pediatric controls or patients with MIS-C or CLLs. DNA is detected by SYTOX green and scale bar is 100 μm. (**E**) NET degradation capabilities were measured in serum or plasma of MIS-C (*n* = 7) and CLL (*n* = 4) samples in the presence or absence of recombinant DNase1. Samples within dashed ovals are those that did not respond to treatment with DNase1. Kruskal-Wallis analysis was performed. (**F**) Representative images of PMA-generated NETs incubated with serum or plasma from patients with MIS-C or CLLs in the presence or absence of recombinant DNase1. DNA is detected by SYTOX green and scale bar is 100 μm; *P < 0.05, **P < 0.01, ***P < 0.001, ****P < 0.0001. RFU, relative fluorescence units.

As circulating NET levels were increased in pediatric patients with CLLs and MIS-C, we tested whether NET degradation mechanisms were impaired in these populations. Control neutrophils were stimulated with phorbol myristate acetate (PMA) for 4 hours to generate NETs, followed by addition of 5% serum or plasma from healthy controls or MIS-C or CLL patients for 16 hours to identify NET degraders versus nondegraders. Italian MIS-C and CLL samples displayed a bimodal distribution, where 7/10 (70%) of MIS-C and 3/27 (11%) of CLL samples tested did not significantly degrade the NETs (Figure 2, A–C). In contrast, 26/27 (96%) of MIS-C samples from Chile and all the pediatric CLL samples from the United States displayed significantly impaired NET degradation when compared with control samples (Figure 2, A–C). Indeed, the degree of NET degradation differed between geographic regions. MIS-C samples from Chile displayed significant impairments in ability to degrade NETs when compared with Italian samples (Figure 2A). Furthermore, the ability to degrade NETs by serum samples from CLL patients from the United States was significantly impaired when compared with those CLL samples obtained from Italy (Figure 2B). These results support that patients with MIS-C and CLLs display impairments in their ability to degrade NETs in vitro in a geographically dependent manner.

To elucidate the possible mechanisms involved in NET degradation impairment of MIS-C and CLL samples, we added exogenous recombinant DNase1 to the sera. We hypothesized that, if the mechanism of impaired NET degradation involved improper function of the endogenous nucleases, adding exogenous nucleases would lead to restoration of the clearance mechanisms. Indeed, addition of exogenous DNase1 to MIS-C or CLL serum samples resulted in a significant increase in NET degradation in most samples when compared with MIS-C or CLL samples in the absence of exogenous DNase1 (Figure 2). However, 2/7 (29%) of the MIS-C samples and 1/4 (25%) of the CLL samples did not exhibit restored clearance capacity with the addition of exogenous nucleases (Figure 2). These results suggest that some patients may have circulating DNase1 inhibitors or other mechanisms that interfere with the ability of nucleases to effectively access the NETs for degradation.

### Impairments in NET degradation in MIS-C and CLL are multifactorial.

Impaired NET degradation can be secondary to various genetic and acquired factors. To test whether genetic factors that may alter the function of nucleases are involved in the impaired NET degradation observed in pediatric COVID-19, we performed whole-genome sequencing association analysis (WGS) within a subset of patients and conducted variant association analysis for genes encoding enzymes or molecules involved in DNA degradation (*DNASE1*, *DNASE1L3*, *DNASE1L1*, *DNASE1L2*, *DNASE2B*, *SERPINB1*, three prime repair exonuclease 1 [*TREX1*], and *TREX2*). The analysis did not reveal any candidate homozygous rare or low-frequency (minor allele frequency [MAF] < 0.1) variants associated with the nondegrader when compared to the degrader group (Supplemental Table 1; supplemental material available online with this article; https://doi.org/10.1172/jci.insight.160332DS1). Furthermore, we did not observe heterozygous common (MAF > 0.1) variants with distribution attributed to the degrader or nondegrader group (Supplemental Table 1). Overall, a targeted genetic association analysis did not produce evidence for associations between genetic alterations in nucleases and impaired NET degradation in the patients. We therefore investigated other factors that may be involved in aberrant NET degradation.

**Figure 3:**
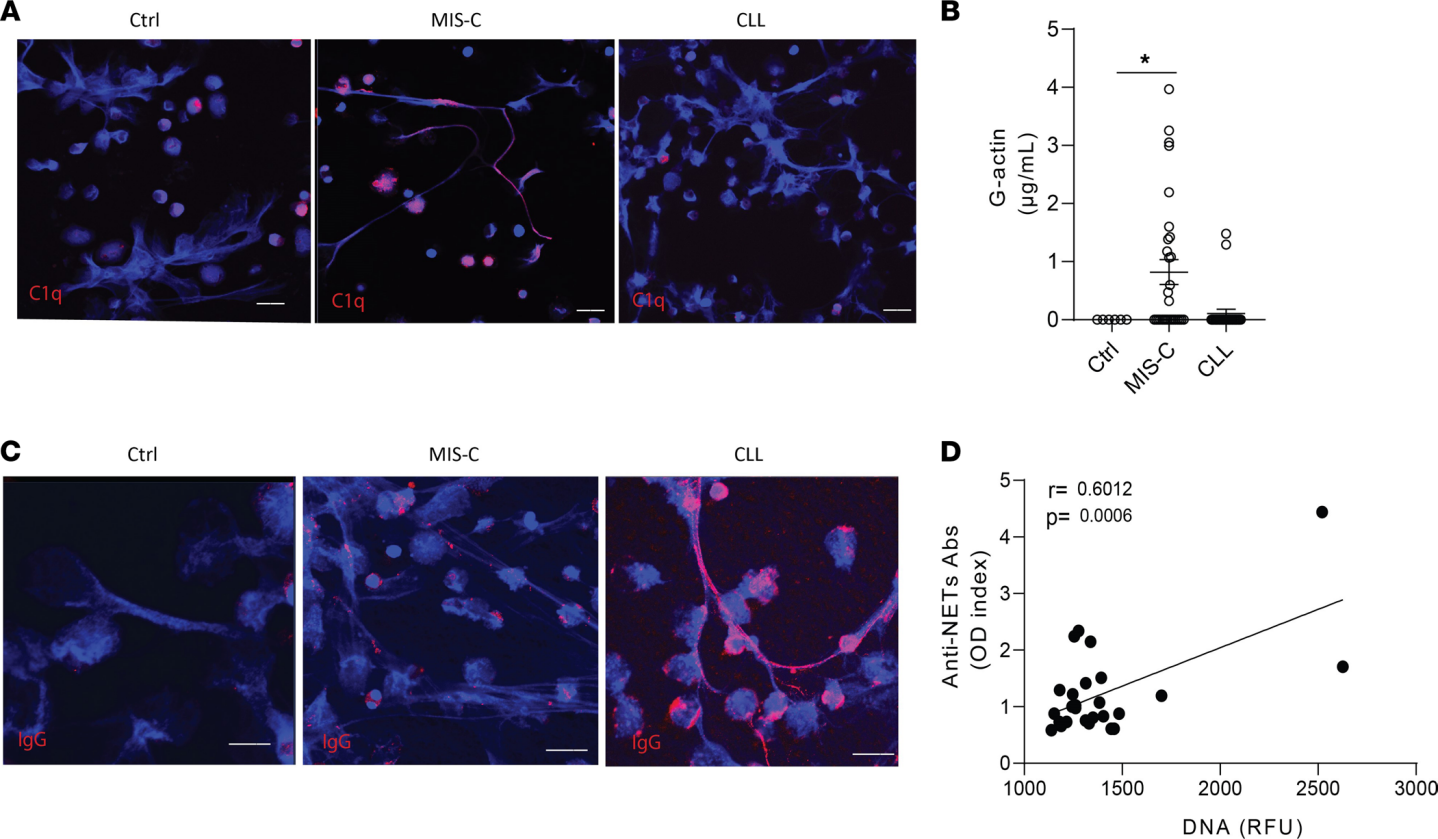
Multiple factors impair NET degradation in MIS-C and CLLs. (**A**) Representative confocal images of immunofluorescence analysis of C1q (red) in PMA-generated NETs by healthy control neutrophils after incubation with serum or plasma from patients with MIS-C or CLLs. DNA is detected in blue and scale bar is 10 μm. (**B**) Levels of circulating G-actin measured in MIS-C (*n* = 27) and CLL (*n* = 26) samples compared with pediatric healthy controls (*n* = 19). Results are the mean ± SEM. Kruskal-Wallis analysis was performed. *P < 0.05. (**C**) Representative confocal images of immunofluorescence analysis of immunoglobulin G (IgG) (red) in PMA-generated NETs after incubation with serum or plasma from patients with MIS-C or CLLs. Scale bar is 10 μm. (**D**) Correlation analysis of levels of anti-NET Abs and degradation capabilities; Pearson analysis was used. Ctrl, controls; OD, optical density; RFU, relative fluorescence units.

Complement activation and deposition of complement factors in NETs have been linked to NET degradation impairments (42, 43). NETs induced in healthy control neutrophils by PMA stimulation stained positive for C1q when they were incubated with serum or plasma from MIS-C, but not from CLL or control, patients (Figure 3A). This observation suggests that complement activation may play a role in impairing NET degradation in MIS-C. G-actin is a natural inhibitor of DNase1 that can impair NET degradation (44). G-actin levels in the samples from the Chilean MIS-C cohort were significantly elevated when compared with healthy controls (Figure 3B) while they were not elevated in CLLs (Figure 3B). Anti-NET autoantibodies (autoAbs) were previously associated with NET degradation impairments in conditions such as systemic lupus erythematosus, and these autoAbs have been reported in adult patients with COVID-19 (44, 45). Therefore, we tested for their presence in MIS-C or CLL samples. We detected IgG binding to NETs in CLL, but not in MIS-C, samples (Figure 3C) in association with impaired degradation (*r* = 0.6012, *P* = 0.0006; Figure 3D). Overall, different factors that include complement activation and increased G-actin levels of and the development of anti-NET Abs were associated with impaired NET degradation in pediatric COVID-19.

### Persistently elevated NET remnants are detected in adult patients with symptomatic COVID-19 in association with disease manifestations.

**Figure 4:**
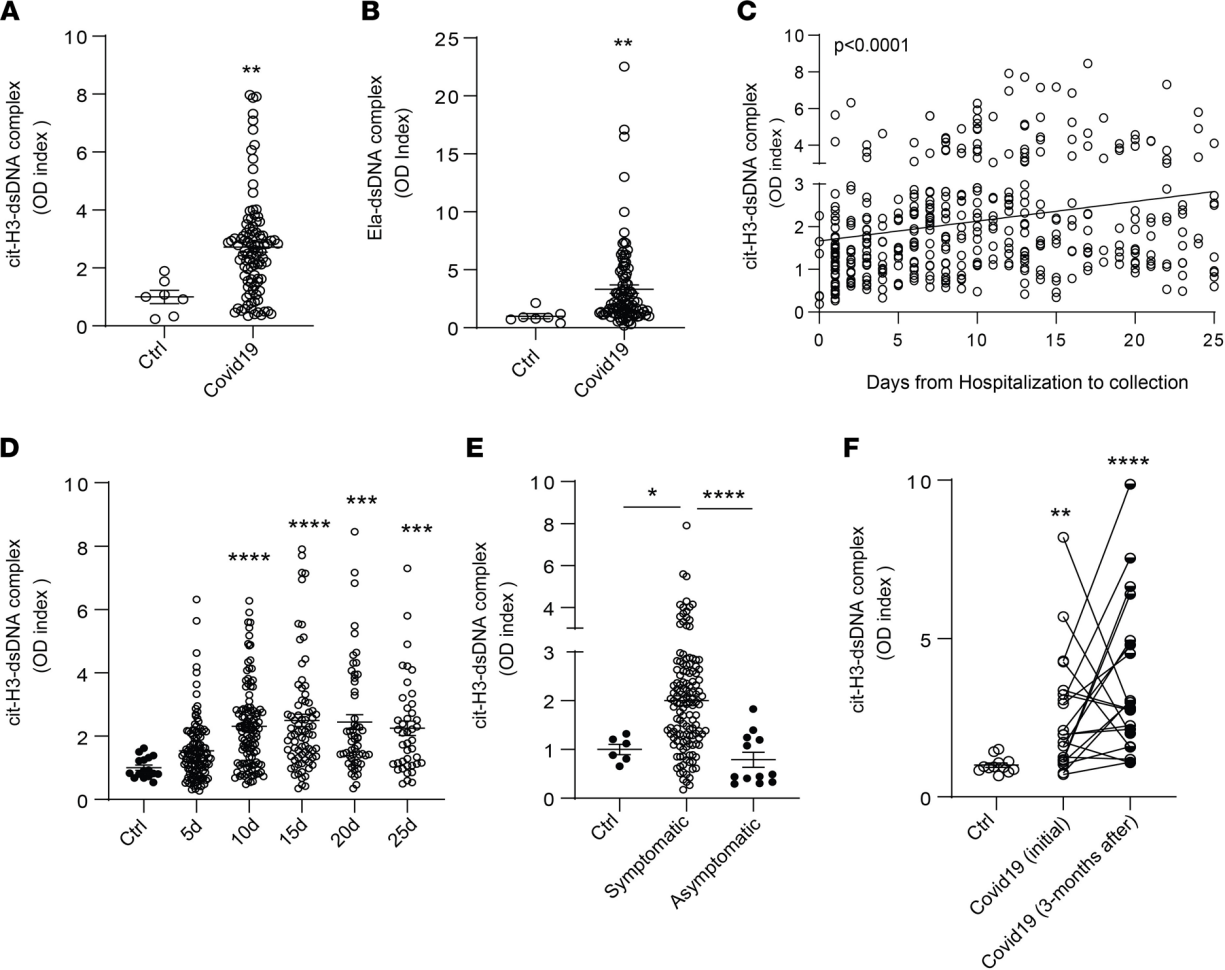
NET remnants are detected in plasma and serum of adult Italian COVID-19 patients. Plasma levels of (**A**) citH3- and (**B**) Ela-dsDNA complexes were measured in adult COVID-19 samples obtained from different cities in Italy (Brescia, Monza, Turin; *n* = 99, control *n* = 7); Mann-Whitney was used. (**C** and **D**) Serum levels of citH3-DNA complexes were elevated in samples obtained up to 25 days (5d *n* = 118, 10d *n* = 117, 15d *n* = 77, 20d *n* = 57, 25d *n* = 42) of hospitalization in samples collected in Brescia, Italy. Kruskal-Wallis analysis was used. Levels of citH3-DNA complexes were measured in (**E**) symptomatic (*n* = 77) and asymptomatic (*n* = 12) COVID-19 samples. (**F**) Levels of citH3-DNA complexes at initial (first 6 days after hospitalization) and 3 months after diagnosis of infection with SARS-CoV-2 (*n* = 20). Results are the mean ± SEM. Kruskal-Wallis analysis was used, *P < 0.05, **P < 0.01, ***P < 0.001, ****P < 0.0001. OD, optical density; ctrl, controls; d, days from diagnosis.

To compare the results obtained in the pediatric population with adults who developed COVID-19, we validated the findings and quantified circulating NETs (citH3-DNA and elastase-DNA complexes) in plasma samples obtained from adult patients admitted to Italian medical centers in Brescia, Turin, and Monza (Table 1). Complexes of citH3-DNA and elastase-DNA were significantly elevated in these patients (Figure 4, A and B). NET complexes were then measured in serum samples collected from Brescia at different days following hospitalization. A significant increase in citH3-DNA complexes was detected in samples collected up to 25 days from the day of hospitalization (Figure 4, C and D). Since infection with SARS-CoV-2 can lead to symptomatic and asymptomatic disease, we asked whether NET complexes would be present in asymptomatic patients infected with SARS-CoV-2. The analysis indicated that circulating NET remnants were significantly lower in asymptomatic when compared with symptomatic patients (Figure 4E). Many COVID-19 patients continue to have clinical complications months after initial diagnosis (46–48). We found that circulating complexes of citH3-DNA were significantly elevated even 3 months after infection diagnosis when compared with healthy controls (Figure 4F). Indeed, there was no significant difference between initial and 3-month postdiagnosis levels of citH3-DNA complexes, although some patients displayed reductions (Figure 4F). These results suggest that increased NET levels in adults with COVID-19 are associated with the presence of symptoms and can persist for several months after initial infection.

### NETs associate with disease severity in adult COVID-19.

**Figure 5:**
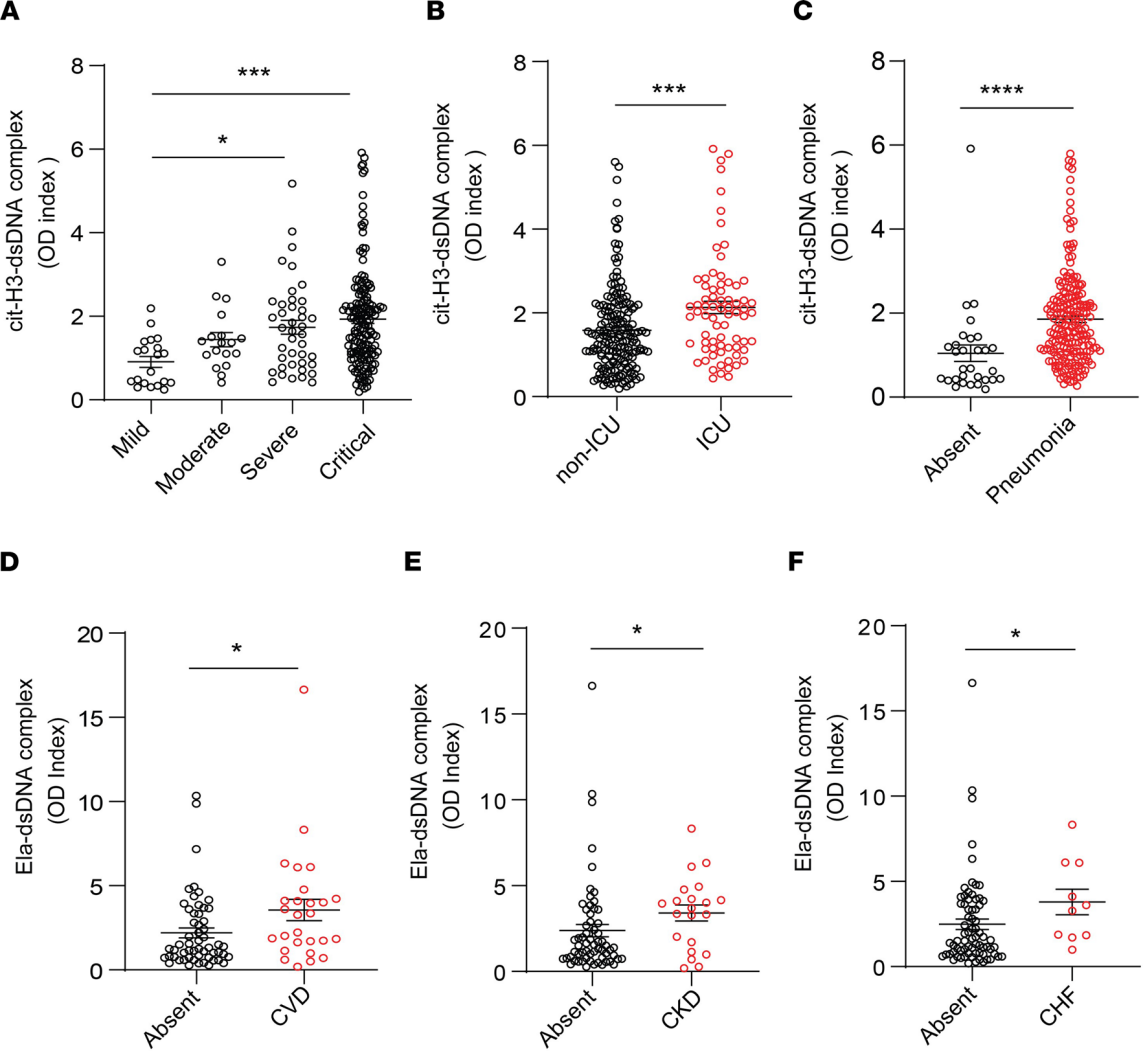
NET remnants are present in plasma and serum of adult COVID-19 patients and correlate with disease severity and comorbidities. Serum levels of (**A**) citH3-DNA complexes collected within the first 5 days after hospitalization were elevated in critical patients (mild *n* = 20, moderate *n* = 18, severe *n* = 41, critical *n* = 156). Kruskal-Wallis analysis was used. Elevated levels of citH3-DNA complexes were detected in (**B**) COVID-19 patients admitted to the intensive care unit (ICU; non-ICU *n* = 175, ICU *n* = 73) and (**C**) those with pneumonia (absent *n* = 30, pneumonia *n* = 203); Mann-Whitney was used. Plasma levels of Ela-DNA complexes were elevated in patients with COVID-19 patients who displayed (**D**) cardiovascular disease (CVD) (absent *n* = 56, CVD *n* = 27), (**E**) chronic kidney disease (CKD) (absent *n* = 62, CKD *n* = 21), and (**F**) congestive heart failure (CHF) (absent *n* = 73, CHF *n* = 10). Results are the mean ± SEM. Mann-Whitney was used. *P < 0.05, ***P < 0.001, ****P < 0.0001. OD, optical density.

To gain further insight into the possible role of NETs in the symptoms and complications of COVID-19, we analyzed multiple parameters that were specifically available for the adult COVID-19 Brescia cohort. Serum levels of citH3-DNA complexes were significantly elevated in patients with COVID-19 who were in severe or critical condition (Figure 5A), required intensive care unit (ICU) admission (Figure 5B), developed pneumonia (Figure 5C), or required high-flow oxygen (Table 2). Serum levels of citH3-DNA were not associated with sex, age, death, diabetes, cardiovascular disease, chronic heart failure, and other comorbidities (Table 2). Serum levels of citH3-DNA were significantly decreased in patients who developed COVID-19 and had underlying chronic hypertension, chronic kidney disease, or history of solid malignancy (Table 2). In the adult patient population with COVID-19, levels of serum citH3-DNA were most consistently associated with disease severity, while levels of elastase-DNA followed less consistent and sometimes opposite trends compared with the citH3-DNA levels (Supplemental Table 2).

Plasma levels of elastase-DNA and citH3-DNA were not associated with sex, age, death, obesity, autoimmune disorder, or neurological-psychiatric disease (Table 3 and Supplemental Table 3). Both plasma elastase-DNA and citH3-DNA levels were associated with having an underlying diagnosis of cardiovascular disease (Figure 5D), chronic kidney disease (Figure 5E), or a history of solid organ transplantation (Table 3 and Supplemental Table 3). Plasma elastase-DNA was also associated with congestive heart failure (Figure 5F), while plasma citH3-DNA was associated with liver disease (Supplemental Table 3). Of note, levels of NET remnants were similar in adult and pediatric COVID-19 cohorts. Overall, levels of NET remnants were associated with specific disease manifestations and disease severity in adults diagnosed with COVID-19.

### NET degradation is impaired in adult patients with COVID-19.

**Figure 6:**
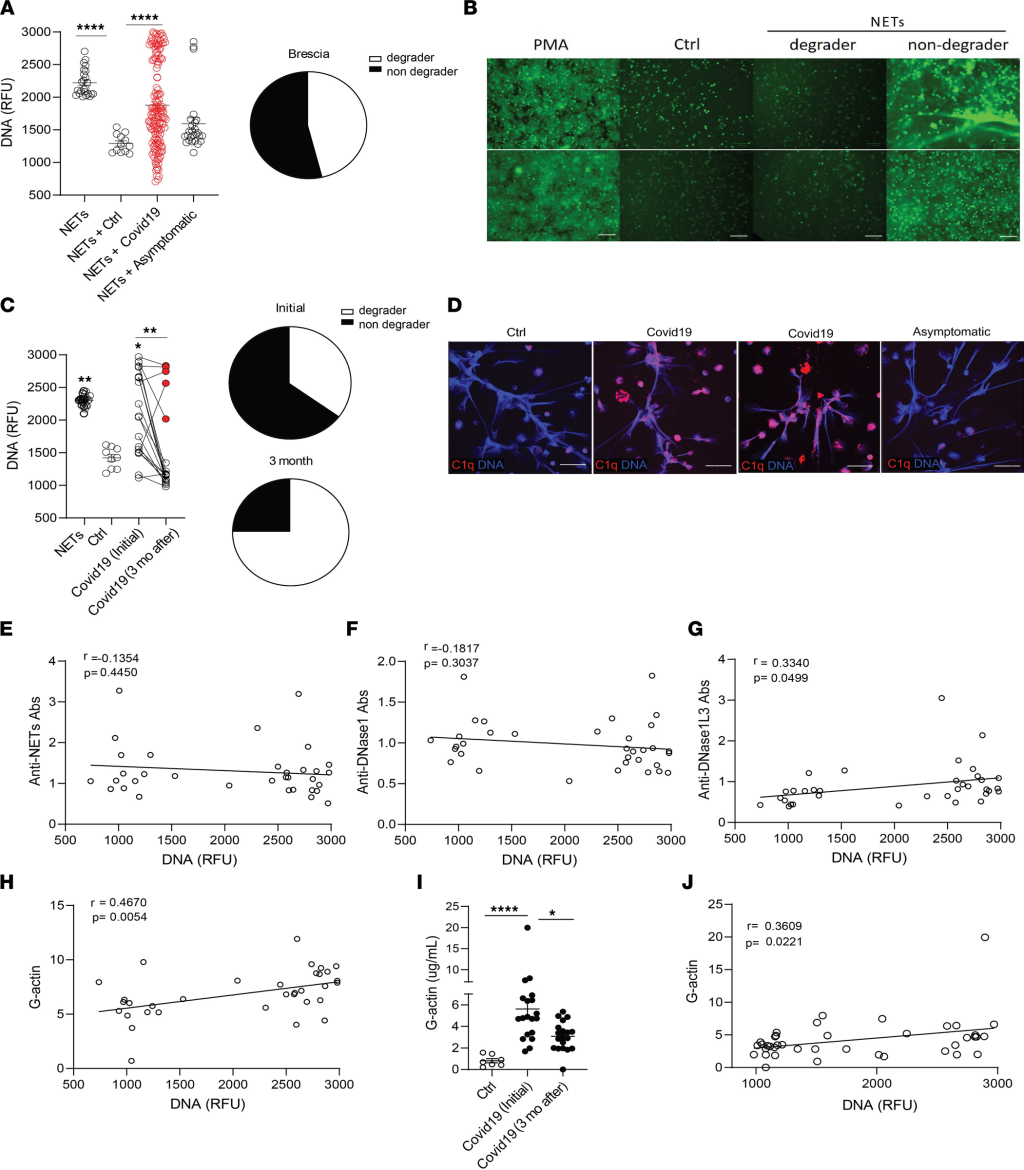
G-actin associates with decreased degradation of NETs in adult Italian COVID-19 patients. (**A**) NET degradation capabilities were measured in COVID-19 serum samples obtained from Brescia, Italy (COVID-19 *n* = 153, asymptomatic *n* = 26, control *n* = 12); pie chart depicts the proportion of degrader (white) and nondegrader (black) serum. Results are the mean ± SEM. Kruskal-Wallis analysis was performed. (**B**) Representative images of PMA generated NETs incubated with serum from controls (*n* = 13) or patients with COVID-19 (*n* = 179). DNA is detected by SYTOX green and scale bar is 100 μm. (**C**) NET degradation capabilities were assessed in 20 patients at initial infection and 3 months after. Pie charts depicting the proportion of degrader (white) and nondegrader (black) at initial and 3 months after infection with SARS-CoV-2. Kruskal-Wallis analysis was performed. (**D**) Representative confocal images of immunofluorescence analysis of C1q deposition (red) in PMA-generated NETs by healthy control neutrophils after incubation with serum from symptomatic and asymptomatic COVID-19 patients. DNA is detected in blue and scale bar is 10 μm. Pearson correlation analysis of (**E**) anti-NET, (**F**) anti-DNase1, and (**G**) anti-DNase1L3 Abs and (**H**) G-actin measured in serum from COVID-19 patients with NET degradation capabilities (DNA). (**I**) Serum G-actin levels in patients with COVID-19 (*n* = 20) at initial and 3 months after diagnosis of infection with SARS-CoV-2. Kruskal-Wallis analysis was performed. (**J**) Pearson correlation analysis of serum G-actin levels in patients with COVID-19 at initial and 3 months after infection with SARS-CoV-2 with NET degradation capabilities (DNA). *P < 0.05, **P < 0.01, ****P < 0.0001; RFU, relative fluorescence units; ctrl, control; mo, months.

We analyzed NET degradation capability of serum samples obtained from Italian adult COVID-19 patients from Brescia. As observed in the pediatric population, impairments in NET degradation were significantly increased in the serum samples from adult COVID-19 patients when compared with healthy controls (Figure 6A), and the impairment was significantly higher in symptomatic COVID-19 patients when compared with those who were asymptomatic at diagnosis (Figure 6A). Indeed, 86/153 (54%) of the adult COVID-19 samples tested displayed impairments in NET degradation (Figure 6, A and B). We investigated whether the lack of NET degradation persisted after the acute infection period had passed. To address this, we tested the NET degradation ability of serum samples from 20 patients with COVID-19 at initial diagnosis and 3 months later. A significant improvement in NET degradation was observed 3 months after initial infection with SARS-CoV-2, with a reduction of nondegraders from 13/20 (65%) to 5/20 (25%) of the samples tested (Figure 6C).

WGS was also performed in adult patients to determine if there were putative genetic contributions of rare or common variants within genes associated with DNA degradation and their link to NET degradation impairments in adult Italian COVID-19 patients. No common (MAF > 0.1) variants were detected with distribution toward degraders or nondegraders. However, a few candidates of loss-of-function homozygous rare (MAF < 0.1) variants and a very rare heterozygous frameshift variant in *DNASE2* were detected in nondegraders (Supplemental Table 4).

As we found evidence of C1q deposition in NETs exposed to MIS-C, but not CLL, we tested if C1q deposition in NETs occurred in adult patients with COVID-19. Confocal microscopy analysis demonstrated C1q deposition in NETs after incubation with symptomatic, but not with control or asymptomatic adult, COVID-19 sera (Figure 6D). Anti-NET autoAbs have been previously reported in adults with COVID-19 (45), and we found them to associate with impaired NET degradation in pediatric CLL samples (Figure 2D). However, we did not find significant levels of anti-NETs autoAbs in the adult COVID-19 samples. Furthermore, levels of anti-NET autoAbs measured in adult COVID-19 samples from Italy did not correlate with impairments in NET degradation (Figure 6E). We quantified autoAbs against the most abundant nucleases in serum: DNase1 and DNase1L3. Levels of autoAbs against DNase1L3, but not against DNase1, were associated with NET degradation impairments (Figure 6, F and G). These results suggest that anti-DNase1L3 Abs may be implicated in impairing clearance of NETs in adult patients with COVID-19. We also found a significant correlation between levels of G-actin and impaired NET degradation in adult COVID-19 serum samples (Figure 6H). Furthermore, longitudinal analysis of patients with COVID-19 demonstrated that G-actin levels were significantly reduced 3 months from initial infection diagnosis (Figure 6I), and these levels significantly correlated (*r* = 0.3609, *P* = 0.0221) with NET degradation impairments (Figure 6J). Like the pediatric samples, these results indicate that the impairments in NET degradation in adults with COVID-19 are multifactorial and may be associated with the presence of autoAbs against DNase1L3 and elevated levels of G-actin.

**Figure 7:**
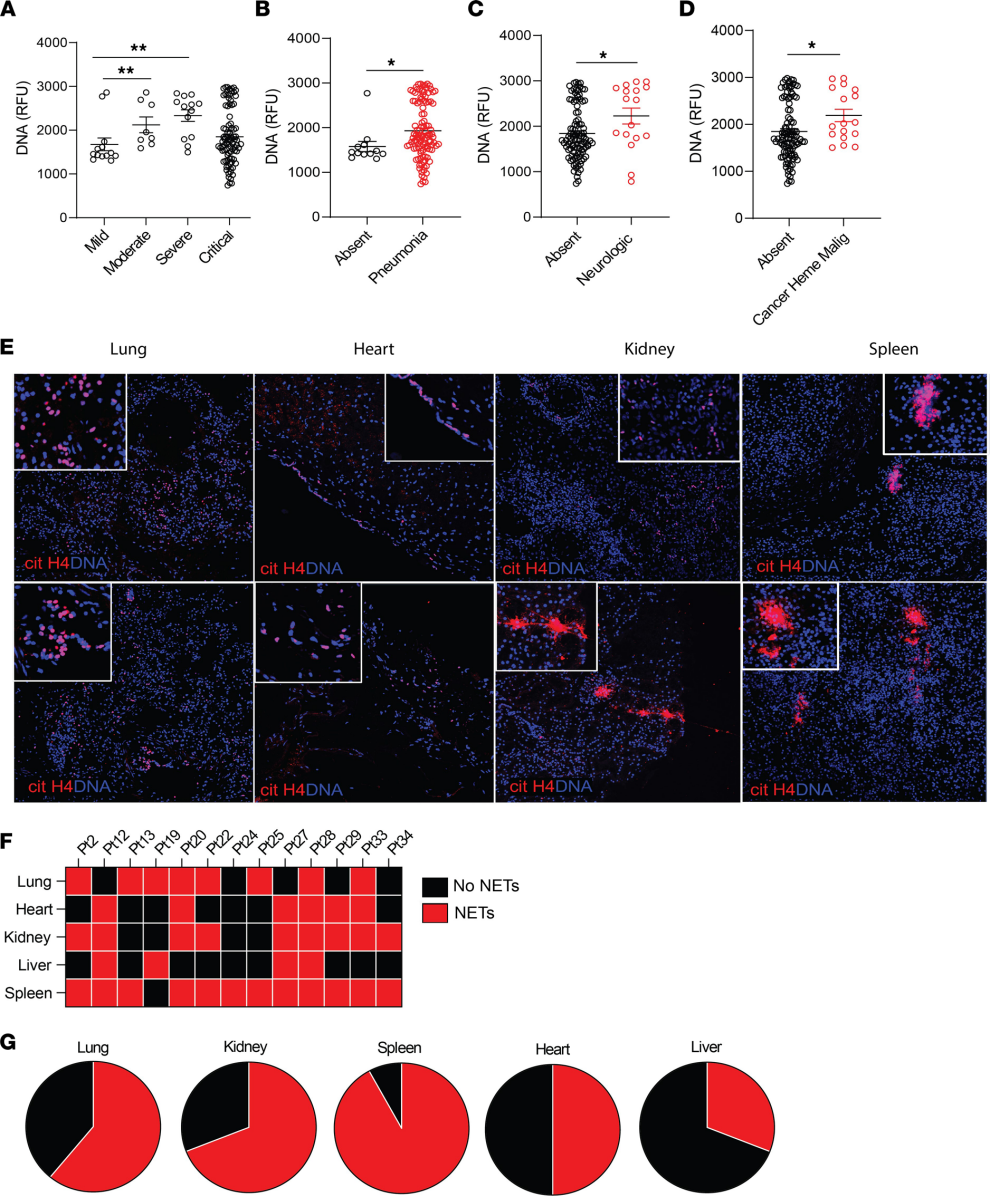
Impairment in NET degradation correlates with comorbidities, and NETs are detected in pulmonary and extrapulmonary tissues in COVID-19. (**A**) Decreased serum NET degradation capabilities in patients with severe COVID-19 from Brescia, Italy (mild *n* = 17, moderate *n* = 8, severe *n* = 13, critical *n* = 89). Kruskal-Wallis analysis was used. Serum samples from COVID-19 patients with (**B**) pneumonia (absent *n* = 17, pneumonia *n* = 109), (**C**) neurological manifestations (absent *n* = 103, neurological *n* = 17), and (**D**) malignancy (absent *n* = 110, malignancy *n* = 17) displayed decreased capabilities of NET degradation. Results are the mean ± SEM. Mann-Whitney was used. (**E**) Representative confocal images of citH4 (red) and DNA (blue) detected in lung, heart, kidney, and spleen tissues obtained from postmortem COVID-19 patients (*n* = 13). Original magnification, ×20, ×40 (insets). (**F**) Summary of tissue NET detection in each patient (*n* = 13). (**G**) Pie charts depicting global NET detection per tissue analyzed. Red indicates positive to citH4 (NETs); black indicates no presence of citH4 signal (no NETs); *P < 0.05, **P < 0.01. RFU, relative fluorescence units.

When assessing associations with COVID-19 manifestations and impaired NET degradation in adults, we found associations with moderate and severe clinical phenotype compared with mild phenotype (Figure 7A and Table 4). In addition, NET degradation was significantly impaired in patients with pneumonia, previous neurological manifestations, and history of cancer (Figure 7, B–D).

### NETs are present in tissues from patients with COVID-19.

Since NETs appear to be associated with various manifestations of COVID-19 and correlate with disease severity, we analyzed if we could detect them in various tissues (lung, heart, spleen, kidney, and liver) available at autopsy from 13 patients who died from disease complications in the United States. We used citH4 marker as a readout of tissue NETs, as described above for the CLL samples. We detected NETs in all these tissues, with variability among patients, including 1 patient who had detectable NETs in all tissues tested (Figure 7E). The organs that displayed more prominent NET infiltrates were the spleen (12/13), kidney (9/13), and lungs (8/13) (Figure 7, F and G), while NET detection in the liver was less prevalent (4/13). In 6 cases, we detected NETs in cardiac tissue (Figure 7F). The characteristics of these patients are displayed in Table 5. These results confirm that NETs are present in various tissues of patients with severe COVID-19.

### Patients with COVID-19 infected with the Omicron variant display decreased NET complexes when compared with infections with other variants.

**Figure 8:**
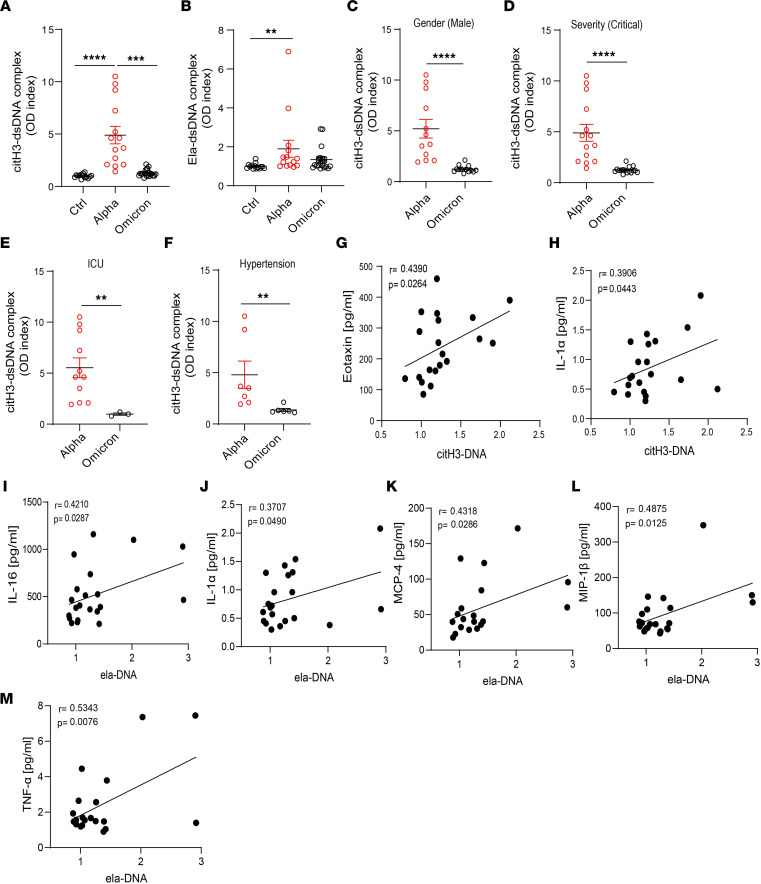
NET remnants are lower in adult unvaccinated patients infected with the Omicron variant. Plasma levels of (**A**) citH3- and (**B**) Ela-dsDNA complexes were measured in Italian COVID-19 patients infected with SARS-CoV-2 Alpha or Omicron variants (ctrl *n* = 14, Alpha *n* = 14, Omicron *n* = 21). Kruskal-Wallis analysis was used. (**C**) Men infected with the Alpha variant of SARS-CoV-2 displayed elevated levels of citH3-DNA complexes (Alpha, *n* = 12, Omicron, *n* = 13). Patients infected with the Omicron variant and with critical severity had (**D**) lower levels of NETs as assessed by decreased levels of plasma citH3-DNA complexes (Alpha *n* = 14, Omicron *n* = 16). (**E**) Patients with COVID-19 in the ICU displayed decreased levels of citH3-DNA complexes when infected with the Omicron variant (Alpha, *n* = 11, Omicron *n* = 3). (**F**) COVID-19 patients with concomitant hypertension displayed decreased levels of citH3-DNA complexes when infected with the Omicron variant (Alpha *n* = 7, Omicron *n* = 6), Mann-Whitney was used. Results are the mean ± SEM. **P < 0.01, ***P < 0.001, ****P < 0.0001. Correlation analysis of citH3-DNA complexes with levels of (**G**) eotaxin or (**H**) IL-1α. Correlation analysis of Ela-DNA complexes with levels of (**I**) IL-16, (**J**) IL-1α, (**K**) MCP-4, (**L**) MIP-1β, or (**M**) TNF-α. Pearson analysis was used. OD, optical density; Ctrl, controls.

During the COVID-19 pandemic, many SARS-CoV-2 variants have emerged (49). The Omicron (B.1.1.529) variant is of particular interest for its rapid spread across the world (50). The Omicron variant harbors 37 mutations in the spike protein (51). Although Omicron is more contagious, there is evidence that indicates that it is milder than other variants (52). We hypothesized that unvaccinated adult patients infected with the Omicron variant would display decreased NET levels when compared with those infected with earlier strains (Alpha). Indeed, citH3-DNA complexes measured in plasma from unvaccinated Italian COVID-19 patients from Brescia infected with the Omicron variant were significantly decreased when compared with levels of those infected with the original strain during the first pandemic peak (Figure 8, A and B). In addition, men infected with the Omicron variant (but not women) displayed significantly lower levels of plasma citH3-DNA complexes when compared with those infected with the original strain (Figure 8C). Levels of citH3-DNA complexes were significantly reduced in patients with the Omicron variant who were in critical condition (Figure 8D), required ICU admission (Figure 8E), had hypertension (Figure 8F), and/or required high-flow oxygen when compared with those infected with the earlier variant. In addition, citH3-DNA levels significantly correlated with Eotaxin (*r* = 0.4390, *P* = 0.0264, Figure 8G) and IL-1α (*r* = 0.3906, *P* = 0.0443, Figure 8H) levels that were quantified in serum samples from the same Omicron-infected Italian patients. Significant correlations were detected between levels of elastase-DNA complexes and levels of IL-16 (*r* = 0.4210, *P* = 0.0287; Figure 8I), IL-1α (*r* = 0.3707, *P* = 0.0490; Figure 8J), monocyte chemoattractant protein-4 (MCP-4) (*r* = 0.4318, *P* = 0.0286; Figure 8K), macrophage inflammatory protein 1β (MIP-1β) (*r* = 0.4875, *P* = 0.0125; Figure 8L), and TNF-α (*r* = 0.5343, *P* = 0.0076; Figure 8M) in these Omicron-infected patients. Overall, these results indicate that different variants of the virus are associated with differences in the levels of circulating NETs and that infection with the Omicron variant is associated with lower levels of these structures when compared with patients infected with earlier strains.

## Discussion

Increasing evidence supports a detrimental role of NETs in COVID-19 pathogenesis. NETs and NET remnants have been detected in adult COVID-19 lung tissues and plasma samples, respectively (30, 38). Our findings expand prior work by demonstrating increased levels of NETs in both MIS-C and CLLs even in the absence of active SARS-CoV-2 infection, as well as an association between circulating NETs and clinical outcomes in pediatric and adult patients from different geographic locations (Supplemental Table 5).

In the present study, we showed that pediatric patients diagnosed with MIS-C and CLL displayed elevations in circulating NETs and in CLL tissue. Impaired NET degradation was also evident and associated with a variety of factors that included complement activation, anti-NET autoAbs, and a natural DNase1 inhibitor, G-actin. As G-actin levels decreased as the disease improved, G-actin induction may be an acute response phenomenon in patients with severe COVID-19 who present with impaired NET degradation. Whether these circulating factors impair NET degradation in vivo remains to be systematically characterized in future studies. Circulating NET levels were associated with comorbidities present in pediatric patients, and there was geographic variability in these findings. Unidentified technical factors associated with sample collection and processing at various centers could, in theory, contribute to these differences. However, the previous observations that SARS-CoV-2 and various COVID-19–associated host factors can directly induce NET formation in this disease (32) support the possibility that environmental or yet-unidentified genetic factors, including SARS-CoV-2 variants, may modulate how neutrophils respond to the infection and associated inflammatory milieu. It will also be important to assess in the future whether the Chilean MIS-C cohort that had higher levels of NETs than the Italian cohort develops stronger associations with autoimmunity features and whether the presence of NETs or NET-associated Abs may show an association with these clinical differences. However, as mentioned in the results section, to date none of the Italian or Chilean patients with MIS-C whom we followed in this study have developed persistent autoimmunity.

NETs are degraded by endogenous DNases that can regulate NET-driven thrombosis (30, 37, 44). Impairments in NET degradation can increase endothelial damage (53) and associate with thrombus formation (32, 37, 54), organ dysfunction, inflammation, and autoimmunity (55). We found that pediatric patients diagnosed with MIS-C or CLLs displayed decreased NET degradation that, in most cases, could be restored with exogenous DNase1. Genetic and environmental factors contribute to decreased DNase1 activity or efficiency. Patients harboring mutations in *DNASE1* are at higher risk of developing lupus (56, 57). However, in the WGS, we did not find genetic drivers of impaired nuclease activity, suggesting that environmental factors, such as differences in microbiome composition, UV light exposure, pollutants, and so on, may be involved. Certainly, assessments of larger cohorts of patients will be required to fully exclude genetic drivers of these abnormalities.

Complement activation, presence of natural DNase1 inhibitors, and autoAbs have been reported to contribute to NET degradation impairments in inflammatory and autoimmune conditions (42, 44, 45). We found that impairments of NET degradation in patients with MIS-C were associated with the presence of the natural DNase 1 inhibitor, G-actin, and with enhanced ability for serum C1q to deposit in NETs. In contrast, anti-NET autoAbs were primarily associated with impairments in NET degradation in patients with CLLs. These results highlight the complexity of neutrophil responses following exposure to SARS-CoV-2 and the associations with different disease manifestations. Addition of exogenous DNase 1 to MIS-C and CLL samples restored NET degradation capabilities in most samples, suggesting that treatment with exogenous DNases could be of benefit to decrease NETs in patients with COVID-19. Notably, aerosolized DNases are being evaluated in clinical trials in COVID-19 (ClinicalTrials.gov identifier: NCT04541979).

Consistent with previous publications, circulating NETs were elevated in adult patients with COVID-19 (38). Samples from symptomatic adults who were diagnosed with COVID-19 during the peak of the pandemic in Italy in early 2020 displayed elevated circulating NETs, while samples from asymptomatic infected patients did not. While the mechanisms promoting enhanced NET formation in COVID-19 remain to be further determined, these results provide a putative link between NETs and symptomatic COVID-19. COVID-19–associated NET formation may involve other mechanisms besides direct infection of neutrophils by SARS-CoV-2; this may explain the lack of NET markers found in infected asymptomatic COVID-19 patients, as well as the evidence for enhanced NET formation in CLL and MIS-C, even in the absence of active documented infection. Furthermore, asymptomatic patients did not display significant impairments in NET degradation or the presence of factors that can interfere with the degradation of the NETs. In contrast, NETs were elevated in symptomatic COVID-19 patients, and they remained elevated at least 3 months after infection diagnosis. Whether this delayed resolution of NET dysregulation contributes to the development and persistence of long COVID symptoms or other chronic complications remains to be determined and should be the focus of future work. Furthermore, NETs were present in extrapulmonary COVID-19 tissues such as heart, liver, kidney, and spleen. These results may implicate NETs in extrapulmonary manifestations seen in patients with COVID-19, even after clearing the virus.

Several SARS-CoV-2 variants have emerged during the COVID-19 pandemic. Omicron is the most recent variant of SARS-CoV-2 reported in late 2021 (50) and was identified as a variant of concern because of its rapid spread (51), despite its association with milder symptoms than other variants (52). Circulating NET levels from adult unvaccinated patients infected with the Omicron variant in Italy were significantly reduced when compared with COVID-19 patients infected with the variant of SARS-CoV-2 that affected Italy early in the pandemic (Figure 8). These results could suggest that lower NET formation induced by this variant may contribute, in part, to the milder symptomatology. Alternatively, the milder inflammatory phenotype induced by this strain may account for downstream effects on neutrophils, limiting their ability to produce NET following this infection. Future studies will be needed to further expand the understanding on how different viral strains modulate neutrophil biology, whether some of these differences explain the geographic variation in NET levels and clearance, and the overall implications of these variations in the reported findings.

Various preexisting comorbidities have been associated (58) with higher morbidity and mortality in adult and pediatric patients who develop COVID-19 (11, 12). In our study, there was no consistent association between specific comorbidities and levels of NETs in adult and pediatric populations. Pericarditis, kidney damage, and neurological and psychiatric manifestations are among the clinical manifestations of a subset of patients with COVID-19 (8, 11, 12, 36). While NETs have been detected in lung samples in COVID-19 and patients with ARDS secondary to other causes (58–64), it remains to be determined whether NETs in extrapulmonary tissues during and after COVID-19 in adults and pediatric patients are associated with prognosis, disease manifestations, or development of long COVID. Here, we were able to detect citH4-positive structures in skin from pediatric patients affected by CLLs and in autopsy tissues from adult patients who died from severe COVID-19. Of note, the lung, kidney, and spleen were the organs with most NETs detected, followed by the heart. Enhanced NET formation or diminished NET clearance may increase the risk of organ damage, and the presence of NETs in these extrapulmonary tissues may link these structures with other complications associated with COVID-19. As the biological significance of NETs during COVID-19 is still not completely elucidated, future studies should investigate the potentially deleterious role of NETs in extrapulmonary tissues that may explain part of the pathophysiology of severe COVID-19.

Limitations of the study are primarily derived from the heterogeneity of the cohorts. The clinical sites did not collect the same clinical characteristics during the peak of the pandemic, limiting in some cases the comparisons among groups, as mentioned above. Furthermore, at this point, we do not have comprehensive details regarding the development of chronic complications and/or long COVID ensuing after COVID-19 infection in these cohorts, which would allow us to further elucidate the impact of enhanced NET formation in this condition. It was difficult to assess the impact of specific therapies on NET formation/degradation as detailed information about medication treatment at each center was not available for our analysis. Additionally, we were limited in the collection of samples from patients infected with different SARS-CoV-2 variants. Finally, the pediatric sample size was small, and future studies will be required to confirm these findings in larger cohorts. Despite these limitations, we were able to work with heterogenous and geographically diverse cohorts in both pediatric and adult populations.

In summary, our findings support a link between NET dysregulation and pediatric manifestations of COVID-19 in association with specific disease manifestations. Pediatric and adult symptomatic COVID-19 patients displayed impaired NET degradation that did not appear to be genetically driven. These results highlight a putative pathogenic role of NETs and impairments in NET degradation in pediatric and adult patients affected by COVID-19.

## Methods

### Sample collection.

Patients fulfilled the CDC’s Health Advisory Case definition for MIS-C, CLLs, and COVID-19. Laboratory confirmation of SARS-CoV-2 infection (positive PCR with/without anti-spike/anti-nucleocapsid serology) was assessed. The severity of the disease in the pediatric and adult COVID-19 cohorts was defined as follows: 1, asymptomatic; 2, mild; 3, moderate; 4, severe; and 5, critical as per the NIH COVID-19 treatment guidelines (65).

Once received in the lab at the local institution, blood samples were immediately centrifuged, and serum/plasma samples were aliquoted and frozen at –20°C, then shipped in dry ice to the NIH. They were thawed only once and processed as described in the appropriate Methods subsection.

For CLL samples obtained from the University of Wisconsin-Madison, patients were prospectively enrolled and consented after the start of the COVID-19 pandemic. Those with prior history of seasonal pernio were included due to speculation for a shared genetic susceptibility with pandemic-associated pernio with other circulating viruses as possible triggers. Affected skin biopsy specimens were obtained from those requiring tissue diagnosis for standard of care. These were formalin-fixed, paraffin-embedded, and sectioned prior to histopathologic diagnosis of CLLs. Laboratory assessments were performed at day 0, at 6–8 weeks, and at 6 months. Samples from healthy individuals without history of COVID-19 symptoms or PCR-confirmed infection were used for serological studies.

The demographic information of controls used for each experiment is included in Supplemental Table 6.

### Postmortem tissue collection.

Autopsies were performed and tissues were collected as described (66) in the NCI’s Laboratory of Pathology at the NIH Clinical Center, coordinated by the NIH COVID-19 Autopsy Consortium and following consent of the legal next of kin.

### WGS.

Genomic DNA was extracted from patients’ whole blood by an automated nucleic acid sample preparation instrument (QIAGEN QIAsymphony SP) using QIAsymphony DNA Midi Kit. DNA samples were then quantified using a fluorescence dye–based assay (PicoGreen dsDNA reagent, Thermo Fisher Scientific) by microplate reader (Molecular Devices SpectraMax Gemini XS). WGS libraries were generated from fragmented DNA using Illumina TruSeq DNA PCR-Free HT Library Preparation Kit with minor modifications for automation (Hamilton STAR Liquid Handling System). Sequencing libraries were quantified using KAPA qPCR Quantification Kit (Roche Light Cycler 480 Instrument II) and combined as 24-plex pools after normalization and sequencing on an Illumina NovaSeq 6000 using an S4 Reagent Kit (300 cycles) using 151 + 8 + 8 + 151 cycle run parameters. Primary sequencing data were demultiplexed using Illumina HAS2.2 pipeline, and sample-level quality control for base quality, coverage, duplicates, and contamination was conducted. All sequencing data were then processed with Burrows–Wheeler Aligner and Genome Analysis Toolkit best-practice pipeline for alignment and variant calling. Whole-genome association data were deposited at dbGaP under accession number phs002245.v1.p1. Data on the genotyped samples are presented in Supplemental Table 7.

### NET complexes ELISA.

A 96-well plate was coated with rabbit polyclonal anti-citH3 (Abcam catalog ab5103) or anti–neutrophil elastase (Calbiochem catalog 481001) at 2.5 μg/mL in PBS overnight at 4°C. Wells were blocked in blocking buffer (1% BSA in PBS) at room temperature (RT) for 1 hour. Plasma or serum were diluted 1:100 in blocking buffer and incubated overnight at 4°C. After washing 3 times with washing buffer (0.05% Tween in PBS), samples were incubated with mouse monoclonal anti–double stranded DNA Ab (MilliporeSigma, clone BV16-13, MAB030) at 1:100 in blocking buffer for 1 hour at RT. The plate was washed 3 times and incubated with goat anti-mouse conjugated HRP Ab (1:10,000) (Bio-Rad, catalog 1706516) for 1 hour at RT. Wells were washed 5 times with 0.05% Tween in PBS followed by addition of 100 μL of TMB substrate (MilliporeSigma) and 50 μL of 0.16 M sulfuric acid stop solution (MilliporeSigma). Absorbance was measured at 450 nm on an ELISA plate reader (Synergy HT, Bio-Tek). ELISA was performed multiple times to ensure reproducibility after freeze-and-thaw cycles and even at high temperature (60°C) for 30 minutes. Data are provided in Supplemental Table 8.

### NET degradation assay.

Control neutrophils were isolated by Ficoll density gradient as described (41). Red blood cells were lysed using hypotonic and hypertonic NaCl buffers. Control neutrophils were resuspended in unsupplemented RPMI (1 × 10^6^ cells/mL) (Thermo Fisher Scientific) and were stimulated with PMA (MilliporeSigma; 500 ng/mL). A total of 100 μL/well was plated in 96-well black plates and incubated for 4 hours at 37°C to induce NETs. Following stimulation, formed NETs were treated with 5% serum from either healthy controls or patients for 16 hours at 37°C. Wells were stained with 0.2 μM SYTOX green (Thermo Fisher Scientific) for 5 minutes. The plate was read using a microplate reader (Synergy HT, Bio-Tek). Results are presented as RFU. Wells were visualized to corroborate the presence or the absence of NETs using the ZOE microscope (Bio-Rad).

### Detection of NETs in tissue specimens.

Parafilm-embedded samples were processed as described (41).

### Immunofluorescence.

Neutrophils were fixed in 4% paraformaldehyde in PBS overnight at 4°C, washed, and blocked with 0.2% porcine gelatin (MilliporeSigma) for 30 minutes, then incubated with primary Ab (anti-C1q, Dako, catalog a0136; or patient serum) for 1 hour in a humid chamber at 37°C. Coverslips were then washed 3 times and incubated 30 minutes with secondary Abs at 37°C: cit-H4 (MilliporeSigma, catalog 07-596), goat anti-rabbit IgG (Invitrogen, catalog A32731), and goat anti-human IgG (Invitrogen, catalog A-11013). Nuclei were counterstained with 1:1000 Hoechst at RT. After washing 3 more times, coverslips were mounted on glass slides using Prolong Gold solution (Invitrogen). Images were acquired on a Zeiss LSM 780 confocal microscope.

### Anti-DNase1, anti-DNase1L3, and NET Ab detection.

A 96-well plate was coated with 2.5 μg/mL of PMA-generated NETs, recombinant DNase1 (Abcam), or DNase1L3 (MyBioSource) in PBS overnight at 4°C. The plate was blocked with 1% BSA for 1 hour, at RT. One microgram of total protein from control or patient skin samples or diluted (1:200) sera were incubated overnight at 4°C. The plate was washed 4 times with 0.05% PBS-Tween (PBS-T). HRP-conjugated anti-human IgG secondary Ab (1:5000; MilliporeSigma, catalog AP309P) was incubated for 1 hour, at RT, followed by 5 washes with PBS-T. The plate was developed in the presence of TMB and read at 450 nm using a microplate reader (Synergy HT, Bio-Tek). Results are presented as OD index (ratio of the OD in the patient serum to the mean OD in healthy control serum).

### G-actin ELISA.

Serum G-actin levels were detected using a commercially available G-Actin ELISA kit (MyBioSource) following the manufacturer’s instructions.

### Measurement of soluble biomarkers.

Cytokine/biomarker analysis was performed on EDTA-treated tube plasma obtained from either patients or healthy volunteers. Duplicate determinations of control samples and samples from healthy volunteers yielded coefficients of variation that were normally less than 20%. Aliquots were stored in a –85°C freezer prior to analysis. Cytokines (IL-1β, IL-1α, IL-2, IL-4, IL-5, IL-6, IL-7, IL-8, IL-10, IL-12p70, IL-12p40, IL-13, IL-15, IL-16, IL-17, IFN-γ, TNF-α, TNF-β, GM-CSF, VEGF, CCL-11/eotaxin-1, CCL26/eotaxin-3, CXCL10/IP-10, MCP-1/CCL2, MCP-4/CCL13, CCL22/MDC, MIP-1α/CCL3, MIP-1β/CCL4, CCL17/TARC) were measured using the V-PLEX Human Cytokine 30-Plex Kit (Meso Scale Discovery) and analyzed on a QuickPlex SQ 120 reader (Meso Scale Discovery) according to the manufacturer’s specifications. Standard curves were analyzed using nonlinear curve fitting, and unknowns were calculated based on the derived equation. Samples that exceeded the highest standards were reanalyzed after dilution until the values fell within the range of the known standards. Two control plasma samples and a control sample spiked with a known quantity of each analyte were analyzed on each plate to assess the interplate variation and to determine the effect of the biological matrix on the measurement of each analyte. For most analytes, the control samples had <25% variation from plate to plate, and the recoveries were generally >70%.

### Statistics.

Data were processed using R version 4.0 and the tidyverse package. All figures were created and associated statistical analyses were performed using GraphPad Prism Version 8.1.1. Mann-Whitney *U* test was used for pairwise comparisons. One-way ANOVA Kruskal-Wallis test (Dunn’s multiple-comparison test) was used to compare parameters among groups. Pearson correlation was used for all noncategorical statistics. All analyses were considered statistically significant at *P* < 0.05.

### Study approval.

All individuals were enrolled in protocols approved by local institutional review boards: Comité Ético Científico Facultad de Medicina Clínica Alemana Universidad del Desarrollo, Santiago, Chile (protocol 2020-41); Comitato Etico Interaziendale A.O.U. Città della Salute e della Scienza di Torino, Turin, Italy (protocol 00282/2020); Ethics Committee of the University of Naples Federico II, Naples, Italy (protocol 158/20); Comitato Etico Provinciale of Brescia, Brescia, Italy (protocol NP-4000); San Gerardo Hospital – University of Milano Bicocca, Monza, Italy; NIAID, NIH (protocols NCT04582903, NCT03394053 and NCT03610802), Bethesda, Maryland, USA; and University of Wisconsin-Madison (protocol 2020-0667), Madison, Wisconsin, USA. All patients gave written informed consent according to all IRBs used.

## Author contributions

CCR designed and performed the experiments, analyzed the data, and wrote the manuscript. TEM and DRC participated in statistical analysis of clinical samples. HCS, YZ, AJO, and CLD performed WGS. KD, RC, HK, DD, MT, OMD, FL, UR, NC, LI, AS, VQ, CF, VD, ALV, LP, AC, MCPH, AB, PB, YE, CA, ERJ, CV, JDLC, RG, CP, JWM, AN, AP, BD, JM, EWC, SEW, MM, AG, MAS, GM, KB, and LDN collected and provided clinical specimens and clinical data, reviewed the manuscript, and provided scientific input. KD, SEW, HCS, DSC, and LDN provided advice and helpful suggestions on project design. JWM, AN, AP, BD, SMH, LMA, and DSC provided tissue specimens and clinical data; DLF and DBK performed cytokine analysis; and MJK planned the project, supervised the work, and wrote the manuscript.

## Supplementary Material

Supplemental data

Supplemental tables 1-8

## Figures and Tables

**Table 5:**
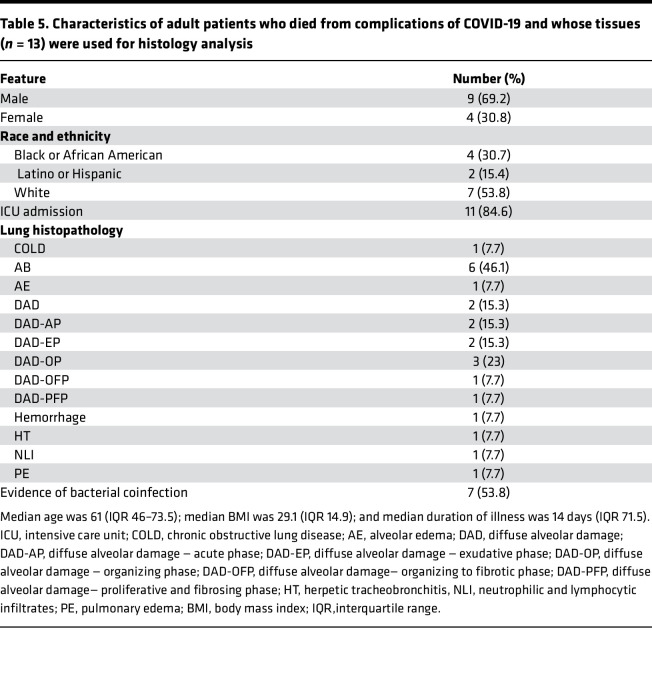
Characteristics of adult patients who died from complications of COVID-19 and whose tissues (*n* = 13) were used for histology analysis

**Table 4:**
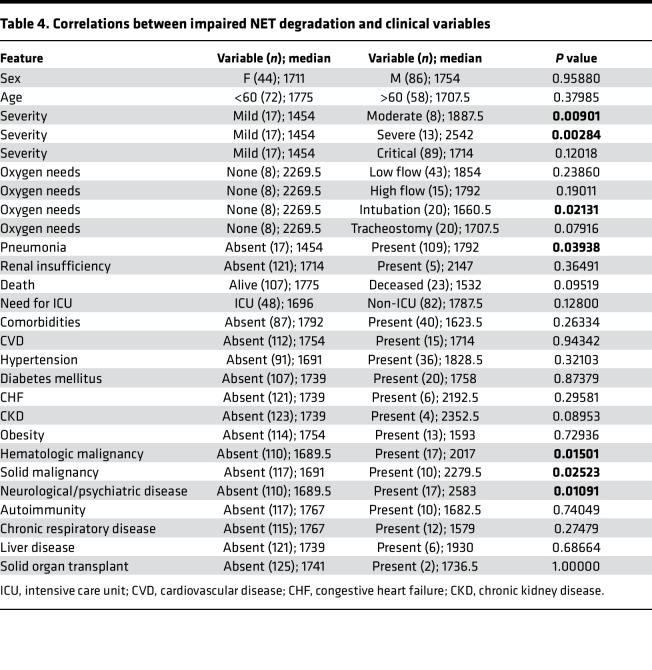
Correlations between impaired NET degradation and clinical variables

**Table 3:**
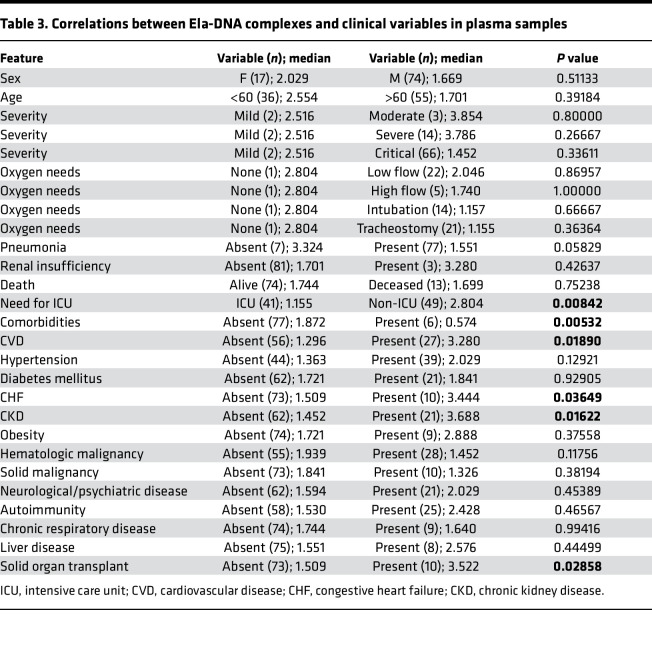
Correlations between Ela-dna complexes and clinical variables in plasma samples

**Table 2:**
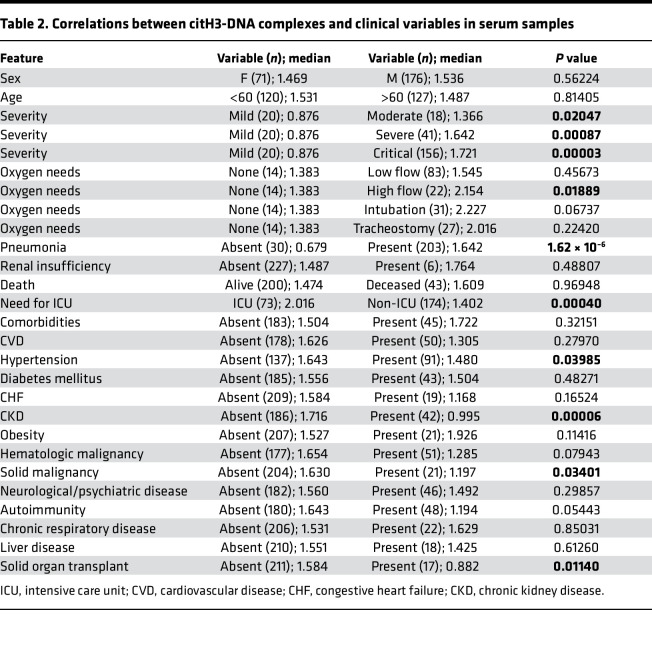
Correlations between citH3-dna complexes and clinical variables in serum samples

**Table 1:**
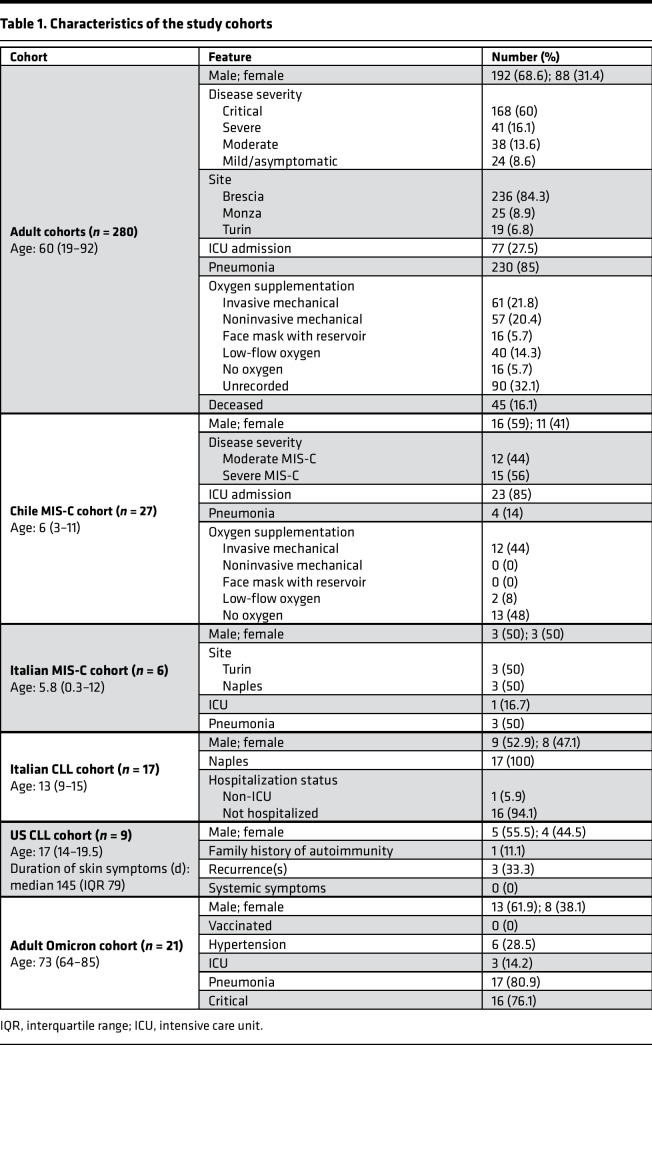
Characteristics of the study cohorts

## Version 1 07/19/2022

In-Press Preview

## Version 2 08/22/2022

Electronic publication

## Version 3 10/05/2022

Updated supplemental materials
