# Supplementary material for: Multicenter analysis of neutrophil extracellular trap dysregulation in adult and pediatric COVID-19
Source: JCI Insight. 2022 Aug 22;7(16):e160332. doi: 10.1172/jci.insight.160332 (PMC9534551; doi:10.1172/jci.insight.160332)
Supplement: Supplemental data [file jciinsight-7-160332-s014.pdf]

*NIH COVID-19 autopsy consortium: Madeline Purcell[1], Nicole Hays[1], Shreya Singireddy[1], Jocelyn Wu[1], Katherine Raja[1], Ryan Curto[1], Shelly J. Curran[2], Sabrina C. Ramelli[2], Mary E. Richert[2], Justin E. Richards[3], John Elder[4], Allen P. Burke[4], Quincy K. Tran[5], Anthony D. Harris[6], Yashvir Sangwan[7], Robert H. Christenson[8], Joseph A. Herrold[9], Ali Tabatabai[9], Eric S. Hochberg[9], Christopher R. Cornachione[9], Andrea R. Levine[9], James W. Eagan Jr. [10], Thomas M. Scalea[11], Douglas Tran[12], Ronson J. Madathil[12], Siamak Dahi[12], Kristopher B. Deatrick[12], Eric M. Krause[12], Kristen Sudano[13], Diane Blume[13], Madhat Arnouk[13], Bethany Radin[13], Michael T. McCurdy[13], Luis J. Perez-Valencia[14], Ashley L. Babyak[14], Sydney R. Stein[14], James M. Dickey[14], Kevin M. Vannella[14], Marcos J. Ramos-Benitez[14], Shahabuddin Soherwardi[15], Kapil K. Saharia[16], Mohammed M. Sajadi[16], Alison Grazioli[17], Willie J. Young[18], Sarah P. Young[18], Billel Gasmi[18], Michelly Sampaio De Melo[18], Sabina Desar[18], Saber Tadros[18], Nadia Nasir[18], Sharika Rajan[18], Esra Dikoglu[18], Neval Ozkaya[18], Stefania Pittaluga[18], Grace Smith, [18] Xueting Jin[18], Joon-Yong Chung[18], Kris Ylaya[18], David E. Kleiner[18], Brian L. Kelsall[19], Elizabeth R. Emanuel[19], Daniel L. Herr[20], Joseph Rabin[20], Justin A. Olivera[21], Megan Blawas[21], Jean E. Chung[22], Amy J. Borth[22], Kimberly A. Bowers[22], Anne M. Weichold[22], Paula A. Minor[22], Emily E. Kelly[22], Mir Ahmad N. Moshref[22].

1. University of Maryland School of Medicine, Baltimore, MD, USA
2. Critical Care Medicine Department, Clinical Center, National Institutes of Health
3. Department of Anesthesiology, Division of Critical Care Medicine, University of Maryland School of Medicine, Baltimore, MD, USA
4. Department of Autopsy and Thoracic Pathology, University of Maryland School of Medicine, Baltimore, MD, USA
5. Department of Emergency Medicine, R. Adams Cowley Shock Trauma Center, University of Maryland School of Medicine, Baltimore, MD, USA

6. Department of Epidemiology and Public Health, University of Maryland School of Medicine, Baltimore, MD, USA
7. Department of Interventional Pulmonology, TidalHealth Peninsula Regional, Salisbury, MD, USA
8. Department of Laboratory Science, University of Maryland School of Medicine, Baltimore, MD, USA
9. Department of Medicine, Division of Pulmonary and Critical Care Medicine, University of Maryland School of Medicine, Baltimore, MD, USA
10. Department of Pathology, University of Maryland, St. Joseph Medical Center, Towson, MD, USA
11. Department of Shock Trauma Critical Care, University of Maryland School of Medicine, Baltimore, MD, USA
12. Department of Surgery, Division of Cardiac Surgery, University of Maryland School of Medicine, Baltimore, MD, USA
13. Division of Critical Care Medicine, Department of Medicine, University of Maryland St. Joseph Medical Center, Towson, MD, USA
14. Emerging Pathogens Section, Critical Care Medicine Department, Clinical Center, National Institutes of Health, Bethesda, MD, USA; Laboratory of Immunoregulation, National Institute of Allergy and Infectious Diseases, Bethesda, MD, USA
15. Hospitalist Department, TidalHealth Peninsula Regional, Salisbury, MD, USA
16. Institute of Human Virology, University of Maryland School of Medicine, Baltimore, MD, USA; Department of Medicine, Division of Pulmonary and Critical Care Medicine, University of Maryland School of Medicine, Baltimore, MD, USA

17. Kidney Disease Section, Kidney Diseases Branch, National Institute of Diabetes and Digestive and Kidney Diseases, National Institutes of Health, Bethesda, MD, USA; Institute of Human Virology, University of Maryland School of Medicine, Baltimore, MD, USA
18. Laboratory of Pathology, Center for Cancer Research, National Cancer Institute, National Institutes of Health, Bethesda, MD, USA
19. Mucosal Immunobiology Section, Laboratory of Molecular Immunology, NIAID, NIH, Bethesda, MD, USA.
20. R Adams Cowley Shock Trauma Center, Department of Medicine and Program in Trauma, University of Maryland School of Medicine, Baltimore, MD, USA
21. Renal Diagnostic and Therapeutics Unit, Kidney Diseases Branch, National Institute of Diabetes and Digestive and Kidney Diseases, National Institutes of Health, Bethesda, MD, USA
22. University of Maryland Medical Center, Baltimore, MD, USA

** COVID-STORM Clinicians: Giuseppe Foti[1,] Giacomo Bellani[1], Giuseppe Citerio[1], Ernesto Contro[1], Fabrizio Luppi[2], Maria Grazia Valsecchi[3], Marina Elena Cazzaniga[4].

[1]Department of Emergency, Anesthesia and Intensive Care, School of Medicine and Surgery, University of Milano-Bicocca, San Gerardo Hospital, Monza, Italy, [2]Department of Pneumology, School of Medicine and Surgery, University of Milano-Bicocca, San Gerardo Hospital, Monza, Italy, [3]Center of Bioinformatics and Biostatistics, School of Medicine and Surgery, University of Milano-Bicocca, San Gerardo Hospital, Monza, Italy, [4]Phase I Research Center, School of Medicine and Surgery, University of Milano-Bicocca, San Gerardo Hospital, Monza, Italy.
