# Supplementary material for: Multicenter analysis of neutrophil extracellular trap dysregulation in adult and pediatric COVID-19
Source: JCI Insight. 2022 Aug 22;7(16):e160332. doi: 10.1172/jci.insight.160332 (PMC9534551; doi:10.1172/jci.insight.160332)
Supplement: Supplemental tables 1-8 [file jciinsight-7-160332-s015.pdf]

SUPPLEMENTARY TABLE 1: WHOLE GENOME SEQUENCING ANALYSIS OF NON DEGRADERS AND DEGRADERS IN PEDIATRIC SAMPLES

AUTOSOMAL DOMINANT

family_id	*Category*	*gene*	*chrom*	*start*	*end*	*ref*	*alt*	*impact*	*impact_severity*	*rs_ids*	*gnomad_MAF*
HGR0001431	NONdegraders-MISC	DNASE1	chr16	3705871	3705872	C	CCCTGGTC	frameshift	HIGH	None	0
HGR0004923	NONdegraders-MISC	DNASE1L3	chr3	58183635	58183636	G	A	missense_	MED	rs3567747	0.0500614
HGR0004914	NONdegraders-MISC	DNASE1	chr16	3707256	3707257	C	T	missense_	MED	rs1483739	0.00109926
HGR0001435	NONdegraders-MISC	DNASE1	chr16	3707256	3707257	C	T	missense_	MED	rs1483739	0.00109926
HGR0004936	NONdegraders-MISC	DNASE2B	chr1	84880426	84880427	G	A	missense_	MED	rs7604239	0.0315103
HGR0001429	Conflict degrader status - M	DNASE1	chr16	3707916	3707917	T	C	missense_	MED	rs7585217	None
HGR0004931	NONdegraders-MISC	DNASE1	chr16	3706648	3706649	G	A	missense_	MED	rs1430585	0.000194037
HGR0004911	NONdegraders-MISC	TREX2	chrX	1.53E+08	1.53E+08	G	A	missense_	MED	rs1412055	0.000233579
HGR0004949	NONdegraders-MISC	DNASE1	chr16	3707022	3707023	C	G	missense_	MED	rs1799891	0.00242734
HGR0004921	NONdegraders-MISC	DNASE1	chr16	3707022	3707023	C	G	missense_	MED	rs1799891	0.00242734
HGR0004939	NONdegraders-MISC	DNASE1	chr16	3707022	3707023	C	G	missense_	MED	rs1799891	0.00242734
HGR0001430	Conflict degrader status - M	DNASE1	chr16	3705478	3705479	G	C	missense_	MED	rs3490739	0.0034904
HGR0001428	Degraders-MISC	DNASE1L1	chrX	1.54E+08	1.54E+08	C	T	missense_	MED	rs3495216	0.0331918
HGR0004914	NONdegraders-MISC	DNASE1L2	chr16	2287214	2287215	G	A	splice_regi	MED	rs160550	0.0886898
HGR0004946	NONdegraders-MISC	DNASE1L2	chr16	2287214	2287215	G	A	splice_regi	MED	rs160550	0.0886898
HGR0004920	NONdegraders-MISC	DNASE1L2	chr16	2287214	2287215	G	A	splice_regi	MED	rs160550	0.0886898
HGR0004905	NONdegraders-MISC	DNASE1L3	chr3	58191229	58191230	G	T	missense_	MED	rs1249194	0.00671661
HGR0004932	NONdegraders-MISC	DNASE1L3	chr3	58191229	58191230	G	T	missense_	MED	rs1249194	0.00671661
HGR0004923	NONdegraders-MISC	DNASE1L3	chr3	58191229	58191230	G	T	missense_	MED	rs1249194	0.00671661
HGR0004931	NONdegraders-MISC	DNASE1L3	chr3	58191229	58191230	G	T	missense_	MED	rs1249194	0.00671661
HGR0004907	NONdegraders-MISC	SERPINB1	chr6	2836485	2836486	T	TA	splice_regi	MED	rs1505646	0.0157305
HGR0001429	Conflict degrader status - M	DNASE1	chr16	3707746	3707747	G	A	missense_	MED	rs1053874	0.444635
HGR0001430	Conflict degrader status - M	DNASE1	chr16	3707746	3707747	G	A	missense_	MED	rs1053874	0.444635
HGR0001428	Degraders-MISC	DNASE1	chr16	3707746	3707747	G	A	missense_	MED	rs1053874	0.444635
HGR0004926	NONdegraders-MISC	DNASE1	chr16	3707746	3707747	G	A	missense_	MED	rs1053874	0.444635
HGR0004927	NONdegraders-MISC	DNASE1	chr16	3707746	3707747	G	A	missense_	MED	rs1053874	0.444635
HGR0004912	NONdegraders-MISC	DNASE1	chr16	3707746	3707747	G	A	missense_	MED	rs1053874	0.444635
HGR0004949	NONdegraders-MISC	DNASE1	chr16	3707746	3707747	G	A	missense_	MED	rs1053874	0.444635
HGR0004914	NONdegraders-MISC	DNASE1	chr16	3707746	3707747	G	A	missense_	MED	rs1053874	0.444635
HGR0001422	NONdegraders-MISC	DNASE1	chr16	3707746	3707747	G	A	missense_	MED	rs1053874	0.444635
HGR0004907	NONdegraders-MISC	DNASE1	chr16	3707746	3707747	G	A	missense_	MED	rs1053874	0.444635
HGR0004911	NONdegraders-MISC	DNASE1	chr16	3707746	3707747	G	A	missense_	MED	rs1053874	0.444635
HGR0001437	NONdegraders-MISC	DNASE1	chr16	3707746	3707747	G	A	missense_	MED	rs1053874	0.444635
HGR0004933	NONdegraders-MISC	DNASE1	chr16	3707746	3707747	G	A	missense_	MED	rs1053874	0.444635
HGR0004909	NONdegraders-MISC	DNASE1	chr16	3707746	3707747	G	A	missense_	MED	rs1053874	0.444635
HGR0004920	NONdegraders-MISC	DNASE1	chr16	3707746	3707747	G	A	missense_	MED	rs1053874	0.444635
HGR0004946	NONdegraders-MISC	DNASE1	chr16	3707746	3707747	G	A	missense_	MED	rs1053874	0.444635
HGR0004930	NONdegraders-MISC	DNASE1	chr16	3707746	3707747	G	A	missense_	MED	rs1053874	0.444635
HGR0004921	NONdegraders-MISC	DNASE1	chr16	3707746	3707747	G	A	missense_	MED	rs1053874	0.444635
HGR0004939	NONdegraders-MISC	DNASE1	chr16	3707746	3707747	G	A	missense_	MED	rs1053874	0.444635
HGR0001424	Degraders-MISC	DNASE2B	chr1	84864255	84864256	G	C	missense_	MED	rs3738573	0.321401
HGR0001436	Degraders-MISC	DNASE2B	chr1	84864255	84864256	G	C	missense_	MED	rs3738573	0.321401
HGR0001434	Degraders-MISC	DNASE2B	chr1	84864255	84864256	G	C	missense_	MED	rs3738573	0.321401
HGR0001432	Degraders-MISC	DNASE2B	chr1	84864255	84864256	G	C	missense_	MED	rs3738573	0.321401
HGR0001433	Degraders-MISC	DNASE2B	chr1	84864255	84864256	G	C	missense_	MED	rs3738573	0.321401
HGR0004926	NONdegraders-MISC	DNASE2B	chr1	84864255	84864256	G	C	missense_	MED	rs3738573	0.321401
HGR0004905	NONdegraders-MISC	DNASE2B	chr1	84864255	84864256	G	C	missense_	MED	rs3738573	0.321401
HGR0004934	NONdegraders-MISC	DNASE2B	chr1	84864255	84864256	G	C	missense_	MED	rs3738573	0.321401
HGR0004932	NONdegraders-MISC	DNASE2B	chr1	84864255	84864256	G	C	missense_	MED	rs3738573	0.321401
HGR0004927	NONdegraders-MISC	DNASE2B	chr1	84864255	84864256	G	C	missense_	MED	rs3738573	0.321401
HGR0004917	NONdegraders-MISC	DNASE2B	chr1	84864255	84864256	G	C	missense_	MED	rs3738573	0.321401
HGR0004923	NONdegraders-MISC	DNASE2B	chr1	84864255	84864256	G	C	missense_	MED	rs3738573	0.321401
HGR0004912	NONdegraders-MISC	DNASE2B	chr1	84864255	84864256	G	C	missense_	MED	rs3738573	0.321401
HGR0004921	NONdegraders-MISC	DNASE2B	chr1	84864255	84864256	G	C	missense_	MED	rs3738573	0.321401
HGR0001435	NONdegraders-MISC	DNASE2B	chr1	84864255	84864256	G	C	missense_	MED	rs3738573	0.321401
HGR0004944	NONdegraders-MISC	DNASE2B	chr1	84864255	84864256	G	C	missense_	MED	rs3738573	0.321401
HGR0004942	NONdegraders-MISC	DNASE2B	chr1	84864255	84864256	G	C	missense_	MED	rs3738573	0.321401
HGR0004931	NONdegraders-MISC	DNASE2B	chr1	84864255	84864256	G	C	missense_	MED	rs3738573	0.321401
HGR0001427	NONdegraders-MISC-repea	DNASE2B	chr1	84864255	84864256	G	C	missense_	MED	rs3738573	0.321401
HGR0001429	Conflict degrader status - M	DNASE2B	chr1	84867609	84867610	G	A	missense_	MED	rs3754274	0.238885
HGR0001434	Degraders-MISC	DNASE2B	chr1	84867609	84867610	G	A	missense_	MED	rs3754274	0.238885
HGR0001424	Degraders-MISC	DNASE2B	chr1	84867609	84867610	G	A	missense_	MED	rs3754274	0.238885
HGR0001432	Degraders-MISC	DNASE2B	chr1	84867609	84867610	G	A	missense_	MED	rs3754274	0.238885
HGR0001433	Degraders-MISC	DNASE2B	chr1	84867609	84867610	G	A	missense_	MED	rs3754274	0.238885
HGR0001436	Degraders-MISC	DNASE2B	chr1	84867609	84867610	G	A	missense_	MED	rs3754274	0.238885
HGR0004912	NONdegraders-MISC	DNASE2B	chr1	84867609	84867610	G	A	missense_	MED	rs3754274	0.238885
HGR0004934	NONdegraders-MISC	DNASE2B	chr1	84867609	84867610	G	A	missense_	MED	rs3754274	0.238885
HGR0004942	NONdegraders-MISC	DNASE2B	chr1	84867609	84867610	G	A	missense_	MED	rs3754274	0.238885
HGR0004923	NONdegraders-MISC	DNASE2B	chr1	84867609	84867610	G	A	missense_	MED	rs3754274	0.238885
HGR0004926	NONdegraders-MISC	DNASE2B	chr1	84867609	84867610	G	A	missense_	MED	rs3754274	0.238885
HGR0004917	NONdegraders-MISC	DNASE2B	chr1	84867609	84867610	G	A	missense_	MED	rs3754274	0.238885
HGR0004932	NONdegraders-MISC	DNASE2B	chr1	84867609	84867610	G	A	missense_	MED	rs3754274	0.238885

HGR0004905	NONdegraders-MISC	DNASE2B	chr1	84867609	84867610	G	A	missense_	MED	rs3754274	0.238885
HGR0004944	NONdegraders-MISC	DNASE2B	chr1	84867609	84867610	G	A	missense_	MED	rs3754274	0.238885
HGR0004927	NONdegraders-MISC	DNASE2B	chr1	84867609	84867610	G	A	missense_	MED	rs3754274	0.238885
HGR0004931	NONdegraders-MISC	DNASE2B	chr1	84867609	84867610	G	A	missense_	MED	rs3754274	0.238885
HGR0001435	NONdegraders-MISC	DNASE2B	chr1	84867609	84867610	G	A	missense_	MED	rs3754274	0.238885
HGR0004921	NONdegraders-MISC	DNASE2B	chr1	84867609	84867610	G	A	missense_	MED	rs3754274	0.238885
HGR0001427	NONdegraders-MISC-repea	DNASE2B	chr1	84867609	84867610	G	A	missense_	MED	rs3754274	0.238885
HGR0001430	Conflict degrader status - M	DNASE1L3	chr3	58186718	58186719	C	A	splice_regi	MED	rs3772985	0.175386
HGR0001436	Degraders-MISC	DNASE1L3	chr3	58186718	58186719	C	A	splice_regi	MED	rs3772985	0.175386
HGR0001425	Degraders-MISC	DNASE1L3	chr3	58186718	58186719	C	A	splice_regi	MED	rs3772985	0.175386
HGR0001434	Degraders-MISC	DNASE1L3	chr3	58186718	58186719	C	A	splice_regi	MED	rs3772985	0.175386
HGR0004933	NONdegraders-MISC	DNASE1L3	chr3	58186718	58186719	C	A	splice_regi	MED	rs3772985	0.175386
HGR0004917	NONdegraders-MISC	DNASE1L3	chr3	58186718	58186719	C	A	splice_regi	MED	rs3772985	0.175386
HGR0004944	NONdegraders-MISC	DNASE1L3	chr3	58186718	58186719	C	A	splice_regi	MED	rs3772985	0.175386
HGR0004949	NONdegraders-MISC	DNASE1L3	chr3	58186718	58186719	C	A	splice_regi	MED	rs3772985	0.175386
HGR0004926	NONdegraders-MISC	DNASE1L3	chr3	58186718	58186719	C	A	splice_regi	MED	rs3772985	0.175386
HGR0004920	NONdegraders-MISC	DNASE1L3	chr3	58186718	58186719	C	A	splice_regi	MED	rs3772985	0.175386
HGR0004910	NONdegraders-MISC	DNASE1L3	chr3	58186718	58186719	C	A	splice_regi	MED	rs3772985	0.175386
HGR0001424	Degraders-MISC	DNASE2B	chr1	84878227	84878228	C	T	splice_regi	MED	rs7511984	0.178255
HGR0001432	Degraders-MISC	DNASE2B	chr1	84878227	84878228	C	T	splice_regi	MED	rs7511984	0.178255
HGR0001422	NONdegraders-MISC	DNASE2B	chr1	84878227	84878228	C	T	splice_regi	MED	rs7511984	0.178255
HGR0004929	NONdegraders-MISC	DNASE2B	chr1	84878227	84878228	C	T	splice_regi	MED	rs7511984	0.178255
HGR0004921	NONdegraders-MISC	DNASE2B	chr1	84878227	84878228	C	T	splice_regi	MED	rs7511984	0.178255

HOMOZYGOUS

family_id	*Category*	*gene*	*chrom*	*start*	*end*	*ref*	*alt*	*impact*	*impact_severity*	*rs_ids*	*gnomad_MAF*
HGR0001425	Degraders-MISC	DNASE2B	chr1	84864255	84864256	G	C	missense_	MED	rs3738573	0.321401
HGR0001425	Degraders-MISC	DNASE2B	chr1	84867609	84867610	G	A	missense_	MED	rs3754274	0.238885
HGR0001423	Degraders-MISC	DNASE2B	chr1	84878227	84878228	C	T	splice_regi	MED	rs7511984	0.178255

COMPOUND HETERO

family_id	*Category*	*gene*	*chrom*	*start*	*end*	*ref*	*alt*	*impact*	*impact_severity*	*rs_ids*	*gnomad_MAF*
HGR0001429	Conflict degrader status - M	DNASE1	chr16	3707746	3707747	G	A	missense_	MED	rs1053874	0.444635
HGR0001429	Conflict degrader status - M	DNASE1	chr16	3707916	3707917	T	C	missense_	MED	rs7585217	None
HGR0001430	Conflict degrader status - M	DNASE1	chr16	3705478	3705479	G	C	missense_	MED	rs3490739	0.0034904
HGR0001430	Conflict degrader status - M	DNASE1	chr16	3707746	3707747	G	A	missense_	MED	rs1053874	0.444635
HGR0004914	NONdegraders-MISC	DNASE1	chr16	3707256	3707257	C	T	missense_	MED	rs1483739	0.00109926
HGR0004914	NONdegraders-MISC	DNASE1	chr16	3707746	3707747	G	A	missense_	MED	rs1053874	0.444635
HGR0004921	NONdegraders-MISC	DNASE1	chr16	3707022	3707023	C	G	missense_	MED	rs1799891	0.00242734
HGR0004921	NONdegraders-MISC	DNASE1	chr16	3707746	3707747	G	A	missense_	MED	rs1053874	0.444635
HGR0004939	NONdegraders-MISC	DNASE1	chr16	3707022	3707023	C	G	missense_	MED	rs1799891	0.00242734
HGR0004939	NONdegraders-MISC	DNASE1	chr16	3707746	3707747	G	A	missense_	MED	rs1053874	0.444635
HGR0004949	NONdegraders-MISC	DNASE1	chr16	3707022	3707023	C	G	missense_	MED	rs1799891	0.00242734
HGR0004949	NONdegraders-MISC	DNASE1	chr16	3707746	3707747	G	A	missense_	MED	rs1053874	0.444635
HGR0001424	Degraders-MISC	DNASE2B	chr1	84878227	84878228	C	T	splice_regi	MED	rs7511984	0.178255
HGR0001424	Degraders-MISC	DNASE2B	chr1	84864255	84864256	G	C	missense_	MED	rs3738573	0.321401
HGR0001424	Degraders-MISC	DNASE2B	chr1	84867609	84867610	G	A	missense_	MED	rs3754274	0.238885
HGR0001432	Degraders-MISC	DNASE2B	chr1	84878227	84878228	C	T	splice_regi	MED	rs7511984	0.178255
HGR0001432	Degraders-MISC	DNASE2B	chr1	84864255	84864256	G	C	missense_	MED	rs3738573	0.321401
HGR0001432	Degraders-MISC	DNASE2B	chr1	84867609	84867610	G	A	missense_	MED	rs3754274	0.238885
HGR0001433	Degraders-MISC	DNASE2B	chr1	84864255	84864256	G	C	missense_	MED	rs3738573	0.321401
HGR0001433	Degraders-MISC	DNASE2B	chr1	84867609	84867610	G	A	missense_	MED	rs3754274	0.238885
HGR0001434	Degraders-MISC	DNASE2B	chr1	84864255	84864256	G	C	missense_	MED	rs3738573	0.321401
HGR0001434	Degraders-MISC	DNASE2B	chr1	84867609	84867610	G	A	missense_	MED	rs3754274	0.238885
HGR0001436	Degraders-MISC	DNASE2B	chr1	84864255	84864256	G	C	missense_	MED	rs3738573	0.321401
HGR0001436	Degraders-MISC	DNASE2B	chr1	84867609	84867610	G	A	missense_	MED	rs3754274	0.238885
HGR0001435	NONdegraders-MISC	DNASE2B	chr1	84864255	84864256	G	C	missense_	MED	rs3738573	0.321401
HGR0001435	NONdegraders-MISC	DNASE2B	chr1	84867609	84867610	G	A	missense_	MED	rs3754274	0.238885
HGR0004905	NONdegraders-MISC	DNASE2B	chr1	84864255	84864256	G	C	missense_	MED	rs3738573	0.321401
HGR0004905	NONdegraders-MISC	DNASE2B	chr1	84867609	84867610	G	A	missense_	MED	rs3754274	0.238885
HGR0004912	NONdegraders-MISC	DNASE2B	chr1	84864255	84864256	G	C	missense_	MED	rs3738573	0.321401
HGR0004912	NONdegraders-MISC	DNASE2B	chr1	84867609	84867610	G	A	missense_	MED	rs3754274	0.238885
HGR0004917	NONdegraders-MISC	DNASE2B	chr1	84864255	84864256	G	C	missense_	MED	rs3738573	0.321401
HGR0004917	NONdegraders-MISC	DNASE2B	chr1	84867609	84867610	G	A	missense_	MED	rs3754274	0.238885
HGR0004921	NONdegraders-MISC	DNASE2B	chr1	84878227	84878228	C	T	splice_regi	MED	rs7511984	0.178255
HGR0004921	NONdegraders-MISC	DNASE2B	chr1	84864255	84864256	G	C	missense_	MED	rs3738573	0.321401
HGR0004921	NONdegraders-MISC	DNASE2B	chr1	84867609	84867610	G	A	missense_	MED	rs3754274	0.238885
HGR0004922	NONdegraders-MISC	DNASE2B	chr1	84864255	84864256	G	C	missense_	MED	rs3738573	0.321401
HGR0004922	NONdegraders-MISC	DNASE2B	chr1	84867609	84867610	G	A	missense_	MED	rs3754274	0.238885
HGR0004923	NONdegraders-MISC	DNASE2B	chr1	84864255	84864256	G	C	missense_	MED	rs3738573	0.321401
HGR0004923	NONdegraders-MISC	DNASE2B	chr1	84867609	84867610	G	A	missense_	MED	rs3754274	0.238885
HGR0004926	NONdegraders-MISC	DNASE2B	chr1	84864255	84864256	G	C	missense_	MED	rs3738573	0.321401
HGR0004926	NONdegraders-MISC	DNASE2B	chr1	84867609	84867610	G	A	missense_	MED	rs3754274	0.238885
HGR0004927	NONdegraders-MISC	DNASE2B	chr1	84864255	84864256	G	C	missense_	MED	rs3738573	0.321401
HGR0004927	NONdegraders-MISC	DNASE2B	chr1	84867609	84867610	G	A	missense_	MED	rs3754274	0.238885
HGR0004931	NONdegraders-MISC	DNASE2B	chr1	84864255	84864256	G	C	missense_	MED	rs3738573	0.321401

HGR0004931	NONdegraders-MISC	DNASE2B	chr1	84867609	84867610	G	A	missense_	MED	rs3754274	0.238885
HGR0004932	NONdegraders-MISC	DNASE2B	chr1	84864255	84864256	G	C	missense_	MED	rs3738573	0.321401
HGR0004932	NONdegraders-MISC	DNASE2B	chr1	84867609	84867610	G	A	missense_	MED	rs3754274	0.238885
HGR0004934	NONdegraders-MISC	DNASE2B	chr1	84864255	84864256	G	C	missense_	MED	rs3738573	0.321401
HGR0004934	NONdegraders-MISC	DNASE2B	chr1	84867609	84867610	G	A	missense_	MED	rs3754274	0.238885
HGR0004942	NONdegraders-MISC	DNASE2B	chr1	84864255	84864256	G	C	missense_	MED	rs3738573	0.321401
HGR0004942	NONdegraders-MISC	DNASE2B	chr1	84867609	84867610	G	A	missense_	MED	rs3754274	0.238885
HGR0004944	NONdegraders-MISC	DNASE2B	chr1	84864255	84864256	G	C	missense_	MED	rs3738573	0.321401
HGR0004944	NONdegraders-MISC	DNASE2B	chr1	84867609	84867610	G	A	missense_	MED	rs3754274	0.238885
HGR0001427	NONdegraders-MISC-repea	DNASE2B	chr1	84864255	84864256	G	C	missense_	MED	rs3738573	0.321401
HGR0001427	NONdegraders-MISC-repea	DNASE2B	chr1	84867609	84867610	G	A	missense_	MED	rs3754274	0.238885

SUPPLEMENTARY TABLE 2: CORRELATIONS BETWEEN ELASTASE: DNA COMPLEXES AND CLINICAL VARIABLES IN SERUM SAMPLES

**FEATURE**	**VARIABLE (N); *MEDIAN***	**VARIABLE (N); MEDIAN**	**P Value**
Sex	F (71); 1.750	M (176); 1.435	0.04329
Age	<60 (120); 1.669	>60 (127); 1.421	0.05880
Severity	Mild (20); 6.740	Moderate (18); 1.392	0.00086
Severity	Mild (20); 6.740	Severe (41); 1.473	2.16E-07
Severity	Mild (20);	Critical (156); 1.405	9.56E-09
Oxygen needs	None (14); 1.737	Low Flow (83); 1.341	0.27880
Oxygen needs	None (14); 1.737	High Flow (22); 1.720	0.85834
Oxygen needs	None (14); 1.737	Intubation (31); 1.489	0.71304
Oxygen needs	None (14); 1.737	Tracheostomy (27); 1.288	0.24261
Pneumonia	Absent (30); 5.596	Present (203); 1.406	4.82E-07
Renal Insufficiency	Absent (227); 1.495	Present (6); 1.423	0.64097
Death	Alive (200); 1.620	Deceased (43); 1.202	0.04125
Need for ICU	ICU (73); 1.404	non-ICU (174); 1.620	0.06302
Comorbidities	Absent (183); 1.406	Present (45); 1.746	0.02078
CVD	Absent (178); 1.529	Present (50); 1.278	0.11870
Hypertension	Absent (137); 1.523	Present (91); 1.357	0.52106
Diabetes Mellitus	Absent (185); 1.495	Present (43); 1.480	0.93352
CHF	Absent (209); 1.489	Present (19); 1.480	0.96089
CKD	Absent (186); 1.500	Present (42); 1.415	0.43488
Obesity	Absent (207); 1.489	Present (21); 1.398	0.95432
Hematologic Malignancy	Absent (177); 1.518	Present (51); 1.404	0.27880
Solid Malignancy	Absent (204); 1.500	Present (24); 1.412	0.68980
Neurologic/Psychiatric Disease	*Absent* (182); 1.488	Present (46); 1.485	0.51216
Autoimmunity	Absent (180); 1.460	Present (48); 1.718	0.15354
Chronic Respiratory Disease	Absent (206); 1.492	Present (22); 1.477	0.42819
Liver Disease	Absent (210); 1.485	Present (18); 1.611	0.99851
Solid Organ Transplant	Absent (211); 1.505	Present (17); 1.357	0.71938

SUPPLEMENTARY TABLE 3: CORRELATIONS BETWEEN CITRULLINATED HISTONE H3: DNA COMPLEXES AND CLINICAL VARIABLES IN PLASMA SAMPLES

FEATURE	VARIABLE (N); *MEDIAN*	VARIABLE (N); MEDIAN	P Value
Sex	F (17); *2.008*	M (73); *1.461*	0.70290
Age	<60 (36); *1.343*	>60 (54); *1.756*	0.55043
Severity	Mild (2); *2.988*	Moderate (3); *3.714*	0.80000
Severity	Mild (2); *2.988*	Severe (14); *2.060*	0.81667
Severity	Mild (2); *2.988*	Critical (65); *1.448*	0.24581
Oxygen needs	None (1); *4.215*	Low Flow (22); *2.032*	0.34783
Oxygen needs	None (1); *4.215*	High Flow (5);*3.254*	0.66667
Oxygen needs	None (1); *4.215*	Intubation (14); *0.902*	0.26667
Oxygen needs	None (1); *4.215*	Tracheostomy (21); *1.013*	0.18182
Pneumonia	Absent (7); *2.934*	Present (77); *1.461*	0.04833
Renal Insufficiency	Absent (81); *1.569*	Present (3); *2.932*	0.51518
Death	Alive (74); *1.458*	Deceased (12); *1.783*	0.67630
Need for ICU	ICU (41); *1.013*	non-ICU (48); *2.260*	0.01554
Comorbidities	Absent (77); *1.752*	Present (6); *0.738*	0.00949
CVD	Absent (56); *1.343*	Present (27); *2.067*	0.03368
Hypertension	Absent (44); *1.076*	Present (39); *2.016*	0.13392
Diabetes Mellitus	Absent (62); *1.489*	Present (21); *1.752*	0.97911
CHF	Absent (73); *1.569*	Present (10); *2.016*	0.47991
CKD	Absent (62); *1.19*	Present (21); *2.481*	0.04486
Obesity	Absent (74); 1.646	Present (9); *1.53*	0.74175
Hematologic Malignancy	Absent (55); *1.752*	Present (28); *1.265*	0.95008
Solid Malignancy	Absent (73); *1.569*	Present (10); *1.764*	0.81745
Neurologic/Psychiatric Disease	*Absent (62); 1.549*	Present (21); *1.752*	0.62620
Autoimmunity	Absent (58); *1.424*	Present (25); *2.008*	0.39607
Chronic Respiratory Disease	Absent (74); *1.646*	Present (9); *1.059*	0.35237
Liver Disease	Absent (75); *1.448*	Present (8); *3.320*	0.01713
Solid Organ Transplant	Absent (73); *1.387*	Present (10); *3.151*	0.00803

ICU: intensive care unit; CVD: cardiovascular disease; CHF: congestive heart failure; CKD: chronic kidney disease.

SUPPLEMENTARY TABLE 4: WHOLE GENOME SEQUENCING ANALYSIS OF NON DEGRADERS AND DEGRADERS IN ADULT SAMPLES

AUTOSOMAL DOMINANT											
HGRepoID	***Category***	***gene***	***chrom***	***start***	***end***	***ref***	***alt***	***impact***	***severity***	***rs_ids***	***gnomad_MAF***
HGR0000051	**NONdegraders-adult**	**DNASE2B**	**chr1**	**84876683**	**84876684**	**T**	**C**	**splice_don**	**HIGH**	**None**	**None**
HGR0000094	**Degraders-adult**	**DNASE2B**	**chr1**	**84876683**	**84876684**	**T**	**C**	**splice_don**	**HIGH**	**None**	**None**
HGR0000085	NONdegraders-adult	DNASE1L3	chr3	58196632	58196633	T	A	start_lost	HIGH	rs142482733	3.23164E-05
HGR0000108	**NONdegraders-adult**	**DNASE2**	**chr19**	**12987121**	**12987123**	**GA**	**G**	**frameshift_**	**HIGH**	**None**	**None**
HGR0001226	Degraders-adult	DNASE1L3	chr3	58183635	58183636	G	A	missense_v	MED	rs35677470	0.0500614
HGR0000028	NONdegraders-adult	DNASE1L3	chr3	58183635	58183636	G	A	missense_v	MED	rs35677470	0.0500614
HGR0000096	Degraders-adult	DNASE1L3	chr3	58183635	58183636	G	A	missense_v	MED	rs35677470	0.0500614
HGR0000058	Degraders-adult	DNASE1L3	chr3	58183635	58183636	G	A	missense_v	MED	rs35677470	0.0500614
HGR0000038	Degraders-adult	DNASE1L3	chr3	58183635	58183636	G	A	missense_v	MED	rs35677470	0.0500614
HGR0000097	**Degraders-adult**	**TREX1**	**chr3**	**48508343**	**48508344**	**G**	**A**	**missense_v**	**MED**	**rs200773268**	**3.23144E-05**
HGR0000014	Degraders-adult	DNASE2B	chr1	84876679	84876680	T	C	missense_v	MED	rs149919241	0.00152056
HGR0001236	Degraders-adult	DNASE2B	chr1	84876679	84876680	T	C	missense_v	MED	rs149919241	0.00152056
HGR0000083	NONdegraders-adult	DNASE1L2	chr16	2287648	2287649	A	C	missense_v	MED	rs62621282	0.00323939
HGR0000082	Degraders-adult	DNASE1L2	chr16	2287648	2287649	A	C	missense_v	MED	rs62621282	0.00323939
HGR0000037	NONdegraders-adult	TREX2	chrX	1.53E+08	1.53E+08	A	G	missense_v	MED	None	None
HGR0000074	NONdegraders-adult	TREX1	chr3	48508747	48508748	A	G	missense_v	MED	None	None
HGR0000094	Degraders-adult	DNASE1L2	chr16	2287264	2287265	C	T	missense_v	MED	rs200934792	0.00042153
HGR0000104	NONdegraders-adult	DNASE1	chr16	3706667	3706668	A	C	missense_v	MED	rs34923865	0.0110644
HGR0000110	Degraders-adult	DNASE1	chr16	3706667	3706668	A	C	missense_v	MED	rs34923865	0.0110644
HGR0000064	Degraders-adult	DNASE1	chr16	3707760	3707761	C	T	missense_v	MED	None	None
HGR0000105	Degraders-adult	DNASE1L2	chr16	2287293	2287294	C	G	missense_v	MED	rs772354278	None
HGR0000056	Degraders-adult	DNASE2	chr19	12987165	12987166	C	T	missense_v	MED	rs766377327	0.000129358
HGR0000006	NONdegraders-adult	DNASE1L3	chr3	58196729	58196730	C	T	splice_regio	MED	None	None
HGR0000095	NONdegraders-adult	DNASE1	chr16	3705478	3705479	G	C	missense_v	MED	rs34907394	0.0034904
HGR0000068	NONdegraders-adult	DNASE1	chr16	3705478	3705479	G	C	missense_v	MED	rs34907394	0.0034904
HGR0000057	NONdegraders-adult	DNASE1	chr16	3705478	3705479	G	C	missense_v	MED	rs34907394	0.0034904
HGR0000110	Degraders-adult	SERPINB1	chr6	2842045	2842046	C	A	splice_regio	MED	None	None
HGR0000081	NONdegraders-adult	DNASE1L3	chr3	58191273	58191274	C	G	missense_v	MED	rs74350392	0.00900581
HGR0000015	NONdegraders-adult	DNASE1L3	chr3	58191273	58191274	C	G	missense_v	MED	rs74350392	0.00900581
HGR0000040	NONdegraders-adult	DNASE1L3	chr3	58191273	58191274	C	G	missense_v	MED	rs74350392	0.00900581
HGR0000028	NONdegraders-adult	DNASE1L3	chr3	58191273	58191274	C	G	missense_v	MED	rs74350392	0.00900581
HGR0001240	NONdegraders-adult	DNASE1L3	chr3	58191273	58191274	C	G	missense_v	MED	rs74350392	0.00900581
HGR0000071	NONdegraders-adult	TREX2	chrX	1.53E+08	1.53E+08	C	T	missense_v	MED	rs200659060	0.00216644
HGR0001235	Degraders-adult	DNASE1	chr16	3705379	3705380	G	T	missense_v	MED	rs8176927	0.0340248
HGR0000079	NONdegraders-adult	DNASE1	chr16	3705379	3705380	G	T	missense_v	MED	rs8176927	0.0340248
HGR0000104	NONdegraders-adult	DNASE1L1	chrX	1.54E+08	1.54E+08	C	T	missense_v	MED	rs34952165	0.0331918
HGR0000059	Degraders-adult	DNASE1L1	chrX	1.54E+08	1.54E+08	C	T	missense_v	MED	rs34952165	0.0331918
HGR0000071	NONdegraders-adult	DNASE1L1	chrX	1.54E+08	1.54E+08	C	T	missense_v	MED	rs34952165	0.0331918
HGR0000104	NONdegraders-adult	DNASE1L2	chr16	2287214	2287215	G	A	splice_regio	MED	rs160550	0.0886898
HGR0000090	NONdegraders-adult	DNASE2	chr19	12991918	12991919	G	C	missense_v	MED	rs112348773	0.00371615
HGR0001229	NONdegraders-adult	SERPINB1	chr6	2834086	2834087	T	C	missense_v	MED	rs759268606	None
HGR0000104	NONdegraders-adult	DNASE1	chr16	3706979	3706980	C	T	missense_v	MED	rs149357200	0.000743903
HGR0000022	NONdegraders-adult	DNASE1L3	chr3	58186751	58186752	G	A	missense_v	MED	rs747122870	None
HGR0000055	NONdegraders-adult	SERPINB1	chr6	2838237	2838241	TAAA	T	splice_regio	MED	rs751822160	9.93772E-05
HGR0001234	Degraders-adult	DNASE1	chr16	3707746	3707747	G	A	missense_v	MED	rs1053874	0.444635
HGR0000097	Degraders-adult	DNASE1	chr16	3707746	3707747	G	A	missense_v	MED	rs1053874	0.444635
HGR0000110	Degraders-adult	DNASE1	chr16	3707746	3707747	G	A	missense_v	MED	rs1053874	0.444635
HGR0000032	Degraders-adult	DNASE1	chr16	3707746	3707747	G	A	missense_v	MED	rs1053874	0.444635
HGR0000087	Degraders-adult	DNASE1	chr16	3707746	3707747	G	A	missense_v	MED	rs1053874	0.444635
HGR0001235	Degraders-adult	DNASE1	chr16	3707746	3707747	G	A	missense_v	MED	rs1053874	0.444635
HGR0001233	Degraders-adult	DNASE1	chr16	3707746	3707747	G	A	missense_v	MED	rs1053874	0.444635
HGR0000107	Degraders-adult	DNASE1	chr16	3707746	3707747	G	A	missense_v	MED	rs1053874	0.444635
HGR0001239	Degraders-adult	DNASE1	chr16	3707746	3707747	G	A	missense_v	MED	rs1053874	0.444635
HGR0000062	Degraders-adult	DNASE1	chr16	3707746	3707747	G	A	missense_v	MED	rs1053874	0.444635
HGR0001237	Degraders-adult	DNASE1	chr16	3707746	3707747	G	A	missense_v	MED	rs1053874	0.444635
HGR0000011	Degraders-adult	DNASE1	chr16	3707746	3707747	G	A	missense_v	MED	rs1053874	0.444635
HGR0001228	Degraders-adult	DNASE1	chr16	3707746	3707747	G	A	missense_v	MED	rs1053874	0.444635
HGR0000059	Degraders-adult	DNASE1	chr16	3707746	3707747	G	A	missense_v	MED	rs1053874	0.444635
HGR0000101	Degraders-adult	DNASE1	chr16	3707746	3707747	G	A	missense_v	MED	rs1053874	0.444635
HGR0001241	Degraders-adult	DNASE1	chr16	3707746	3707747	G	A	missense_v	MED	rs1053874	0.444635
HGR0000007	Degraders-adult	DNASE1	chr16	3707746	3707747	G	A	missense_v	MED	rs1053874	0.444635
HGR0000026	Degraders-adult	DNASE1	chr16	3707746	3707747	G	A	missense_v	MED	rs1053874	0.444635
HGR0000061	Degraders-adult	DNASE1	chr16	3707746	3707747	G	A	missense_v	MED	rs1053874	0.444635
HGR0000038	Degraders-adult	DNASE1	chr16	3707746	3707747	G	A	missense_v	MED	rs1053874	0.444635
HGR0001224	Degraders-adult	DNASE1	chr16	3707746	3707747	G	A	missense_v	MED	rs1053874	0.444635

HGR0000014	Degraders-adult	DNASE1	chr16	3707746	3707747	G	A	missense_v	MED	rs1053874	0.444635
HGR0001236	Degraders-adult	DNASE1	chr16	3707746	3707747	G	A	missense_v	MED	rs1053874	0.444635
HGR0000020	NONdegraders-adult	DNASE1	chr16	3707746	3707747	G	A	missense_v	MED	rs1053874	0.444635
HGR0000025	NONdegraders-adult	DNASE1	chr16	3707746	3707747	G	A	missense_v	MED	rs1053874	0.444635
HGR0000088	NONdegraders-adult	DNASE1	chr16	3707746	3707747	G	A	missense_v	MED	rs1053874	0.444635
HGR0000024	NONdegraders-adult	DNASE1	chr16	3707746	3707747	G	A	missense_v	MED	rs1053874	0.444635
HGR0000051	NONdegraders-adult	DNASE1	chr16	3707746	3707747	G	A	missense_v	MED	rs1053874	0.444635
HGR0000002	NONdegraders-adult	DNASE1	chr16	3707746	3707747	G	A	missense_v	MED	rs1053874	0.444635
HGR0000055	NONdegraders-adult	DNASE1	chr16	3707746	3707747	G	A	missense_v	MED	rs1053874	0.444635
HGR0000104	NONdegraders-adult	DNASE1	chr16	3707746	3707747	G	A	missense_v	MED	rs1053874	0.444635
HGR0000044	NONdegraders-adult	DNASE1	chr16	3707746	3707747	G	A	missense_v	MED	rs1053874	0.444635
HGR0000053	NONdegraders-adult	DNASE1	chr16	3707746	3707747	G	A	missense_v	MED	rs1053874	0.444635
HGR0000008	NONdegraders-adult	DNASE1	chr16	3707746	3707747	G	A	missense_v	MED	rs1053874	0.444635
HGR0000047	NONdegraders-adult	DNASE1	chr16	3707746	3707747	G	A	missense_v	MED	rs1053874	0.444635
HGR0000004	NONdegraders-adult	DNASE1	chr16	3707746	3707747	G	A	missense_v	MED	rs1053874	0.444635
HGR0000078	NONdegraders-adult	DNASE1	chr16	3707746	3707747	G	A	missense_v	MED	rs1053874	0.444635
HGR0000065	NONdegraders-adult	DNASE1	chr16	3707746	3707747	G	A	missense_v	MED	rs1053874	0.444635
HGR0000109	NONdegraders-adult	DNASE1	chr16	3707746	3707747	G	A	missense_v	MED	rs1053874	0.444635
HGR0000041	NONdegraders-adult	DNASE1	chr16	3707746	3707747	G	A	missense_v	MED	rs1053874	0.444635
HGR0000006	NONdegraders-adult	DNASE1	chr16	3707746	3707747	G	A	missense_v	MED	rs1053874	0.444635
HGR0000093	NONdegraders-adult	DNASE1	chr16	3707746	3707747	G	A	missense_v	MED	rs1053874	0.444635
HGR0000040	NONdegraders-adult	DNASE1	chr16	3707746	3707747	G	A	missense_v	MED	rs1053874	0.444635
HGR0000021	NONdegraders-adult	DNASE1	chr16	3707746	3707747	G	A	missense_v	MED	rs1053874	0.444635
HGR0001228	Degraders-adult	DNASE2B	chr1	84864255	84864256	G	C	missense_v	MED	rs3738573	0.321401
HGR0001236	Degraders-adult	DNASE2B	chr1	84864255	84864256	G	C	missense_v	MED	rs3738573	0.321401
HGR0000038	Degraders-adult	DNASE2B	chr1	84864255	84864256	G	C	missense_v	MED	rs3738573	0.321401
HGR0000056	Degraders-adult	DNASE2B	chr1	84864255	84864256	G	C	missense_v	MED	rs3738573	0.321401
HGR0000014	Degraders-adult	DNASE2B	chr1	84864255	84864256	G	C	missense_v	MED	rs3738573	0.321401
HGR0000082	Degraders-adult	DNASE2B	chr1	84864255	84864256	G	C	missense_v	MED	rs3738573	0.321401
HGR0000101	Degraders-adult	DNASE2B	chr1	84864255	84864256	G	C	missense_v	MED	rs3738573	0.321401
HGR0001226	Degraders-adult	DNASE2B	chr1	84864255	84864256	G	C	missense_v	MED	rs3738573	0.321401
HGR0000023	Degraders-adult	DNASE2B	chr1	84864255	84864256	G	C	missense_v	MED	rs3738573	0.321401
HGR0000062	Degraders-adult	DNASE2B	chr1	84864255	84864256	G	C	missense_v	MED	rs3738573	0.321401
HGR0000013	Degraders-adult	DNASE2B	chr1	84864255	84864256	G	C	missense_v	MED	rs3738573	0.321401
HGR0000054	Degraders-adult	DNASE2B	chr1	84864255	84864256	G	C	missense_v	MED	rs3738573	0.321401
HGR0000009	Degraders-adult	DNASE2B	chr1	84864255	84864256	G	C	missense_v	MED	rs3738573	0.321401
HGR0000061	Degraders-adult	DNASE2B	chr1	84864255	84864256	G	C	missense_v	MED	rs3738573	0.321401
HGR0000002	NONdegraders-adult	DNASE2B	chr1	84864255	84864256	G	C	missense_v	MED	rs3738573	0.321401
HGR0000083	NONdegraders-adult	DNASE2B	chr1	84864255	84864256	G	C	missense_v	MED	rs3738573	0.321401
HGR0000008	NONdegraders-adult	DNASE2B	chr1	84864255	84864256	G	C	missense_v	MED	rs3738573	0.321401
HGR0000003	NONdegraders-adult	DNASE2B	chr1	84864255	84864256	G	C	missense_v	MED	rs3738573	0.321401
HGR0000069	NONdegraders-adult	DNASE2B	chr1	84864255	84864256	G	C	missense_v	MED	rs3738573	0.321401
HGR0000050	NONdegraders-adult	DNASE2B	chr1	84864255	84864256	G	C	missense_v	MED	rs3738573	0.321401
HGR0000091	NONdegraders-adult	DNASE2B	chr1	84864255	84864256	G	C	missense_v	MED	rs3738573	0.321401
HGR0000086	NONdegraders-adult	DNASE2B	chr1	84864255	84864256	G	C	missense_v	MED	rs3738573	0.321401
HGR0000037	NONdegraders-adult	DNASE2B	chr1	84864255	84864256	G	C	missense_v	MED	rs3738573	0.321401
HGR0000044	NONdegraders-adult	DNASE2B	chr1	84864255	84864256	G	C	missense_v	MED	rs3738573	0.321401
HGR0000019	NONdegraders-adult	DNASE2B	chr1	84864255	84864256	G	C	missense_v	MED	rs3738573	0.321401
HGR0000078	NONdegraders-adult	DNASE2B	chr1	84864255	84864256	G	C	missense_v	MED	rs3738573	0.321401
HGR0000080	NONdegraders-adult	DNASE2B	chr1	84864255	84864256	G	C	missense_v	MED	rs3738573	0.321401
HGR0000065	NONdegraders-adult	DNASE2B	chr1	84864255	84864256	G	C	missense_v	MED	rs3738573	0.321401
HGR0000079	NONdegraders-adult	DNASE2B	chr1	84864255	84864256	G	C	missense_v	MED	rs3738573	0.321401
HGR0000024	NONdegraders-adult	DNASE2B	chr1	84864255	84864256	G	C	missense_v	MED	rs3738573	0.321401
HGR0000104	NONdegraders-adult	DNASE2B	chr1	84864255	84864256	G	C	missense_v	MED	rs3738573	0.321401
HGR0001227	NONdegraders-adult	DNASE2B	chr1	84864255	84864256	G	C	missense_v	MED	rs3738573	0.321401
HGR0000074	NONdegraders-adult	DNASE2B	chr1	84864255	84864256	G	C	missense_v	MED	rs3738573	0.321401
HGR0000016	NONdegraders-adult	DNASE2B	chr1	84864255	84864256	G	C	missense_v	MED	rs3738573	0.321401
HGR0000098	NONdegraders-adult	DNASE2B	chr1	84864255	84864256	G	C	missense_v	MED	rs3738573	0.321401
HGR0000033	NONdegraders-adult	DNASE2B	chr1	84864255	84864256	G	C	missense_v	MED	rs3738573	0.321401
HGR0000073	NONdegraders-adult	DNASE2B	chr1	84864255	84864256	G	C	missense_v	MED	rs3738573	0.321401
HGR0000055	NONdegraders-adult	DNASE2B	chr1	84864255	84864256	G	C	missense_v	MED	rs3738573	0.321401
HGR0000092	NONdegraders-adult	DNASE2B	chr1	84864255	84864256	G	C	missense_v	MED	rs3738573	0.321401
HGR0000068	NONdegraders-adult	DNASE2B	chr1	84864255	84864256	G	C	missense_v	MED	rs3738573	0.321401
HGR0000030	NONdegraders-adult	DNASE2B	chr1	84864255	84864256	G	C	missense_v	MED	rs3738573	0.321401
HGR0000020	NONdegraders-adult	DNASE2B	chr1	84864255	84864256	G	C	missense_v	MED	rs3738573	0.321401
HGR0000088	NONdegraders-adult	DNASE2B	chr1	84864255	84864256	G	C	missense_v	MED	rs3738573	0.321401
HGR0000077	NONdegraders-adult	DNASE2B	chr1	84864255	84864256	G	C	missense_v	MED	rs3738573	0.321401
HGR0000085	NONdegraders-adult	DNASE2B	chr1	84864255	84864256	G	C	missense_v	MED	rs3738573	0.321401
HGR0000071	NONdegraders-adult	DNASE2B	chr1	84864255	84864256	G	C	missense_v	MED	rs3738573	0.321401
HGR0000034	NONdegraders-adult	DNASE2B	chr1	84864255	84864256	G	C	missense_v	MED	rs3738573	0.321401
HGR0000089	NONdegraders-adult	DNASE2B	chr1	84864255	84864256	G	C	missense_v	MED	rs3738573	0.321401
HGR0000047	NONdegraders-adult	DNASE2B	chr1	84864255	84864256	G	C	missense_v	MED	rs3738573	0.321401

HGR0000099	NONdegraders-adult	DNASE2B	chr1	84864255	84864256	G	C	missense_v	MED	rs3738573	0.321401
HGR0000081	NONdegraders-adult	DNASE2B	chr1	84864255	84864256	G	C	missense_v	MED	rs3738573	0.321401
HGR0000106	NONdegraders-adult	DNASE2B	chr1	84864255	84864256	G	C	missense_v	MED	rs3738573	0.321401
HGR0001230	NONdegraders-adult	DNASE2B	chr1	84864255	84864256	G	C	missense_v	MED	rs3738573	0.321401
HGR0000006	NONdegraders-adult	DNASE2B	chr1	84864255	84864256	G	C	missense_v	MED	rs3738573	0.321401
HGR0000109	NONdegraders-adult	DNASE2B	chr1	84864255	84864256	G	C	missense_v	MED	rs3738573	0.321401
HGR0000040	NONdegraders-adult	DNASE2B	chr1	84864255	84864256	G	C	missense_v	MED	rs3738573	0.321401
HGR0000036	NONdegraders-adult	DNASE2B	chr1	84864255	84864256	G	C	missense_v	MED	rs3738573	0.321401
HGR0000067	NONdegraders-adult	DNASE2B	chr1	84864255	84864256	G	C	missense_v	MED	rs3738573	0.321401
HGR0000075	NONdegraders-adult	DNASE2B	chr1	84864255	84864256	G	C	missense_v	MED	rs3738573	0.321401
HGR0000063	NONdegraders-adult	DNASE2B	chr1	84864255	84864256	G	C	missense_v	MED	rs3738573	0.321401
HGR0000090	NONdegraders-adult	DNASE2B	chr1	84864255	84864256	G	C	missense_v	MED	rs3738573	0.321401
HGR0000017	NONdegraders-adult	DNASE2B	chr1	84864255	84864256	G	C	missense_v	MED	rs3738573	0.321401
HGR0001240	NONdegraders-adult	DNASE2B	chr1	84864255	84864256	G	C	missense_v	MED	rs3738573	0.321401
HGR0000056	Degraders-adult	DNASE2B	chr1	84867609	84867610	G	A	missense_v	MED	rs3754274	0.238885
HGR0000023	Degraders-adult	DNASE2B	chr1	84867609	84867610	G	A	missense_v	MED	rs3754274	0.238885
HGR0001234	Degraders-adult	DNASE2B	chr1	84867609	84867610	G	A	missense_v	MED	rs3754274	0.238885
HGR0000082	Degraders-adult	DNASE2B	chr1	84867609	84867610	G	A	missense_v	MED	rs3754274	0.238885
HGR0001228	Degraders-adult	DNASE2B	chr1	84867609	84867610	G	A	missense_v	MED	rs3754274	0.238885
HGR0000001	Degraders-adult	DNASE2B	chr1	84867609	84867610	G	A	missense_v	MED	rs3754274	0.238885
HGR0000038	Degraders-adult	DNASE2B	chr1	84867609	84867610	G	A	missense_v	MED	rs3754274	0.238885
HGR0000061	Degraders-adult	DNASE2B	chr1	84867609	84867610	G	A	missense_v	MED	rs3754274	0.238885
HGR0000062	Degraders-adult	DNASE2B	chr1	84867609	84867610	G	A	missense_v	MED	rs3754274	0.238885
HGR0000054	Degraders-adult	DNASE2B	chr1	84867609	84867610	G	A	missense_v	MED	rs3754274	0.238885
HGR0000101	Degraders-adult	DNASE2B	chr1	84867609	84867610	G	A	missense_v	MED	rs3754274	0.238885
HGR0000013	Degraders-adult	DNASE2B	chr1	84867609	84867610	G	A	missense_v	MED	rs3754274	0.238885
HGR0000060	Degraders-adult	DNASE2B	chr1	84867609	84867610	G	A	missense_v	MED	rs3754274	0.238885
HGR0000009	Degraders-adult	DNASE2B	chr1	84867609	84867610	G	A	missense_v	MED	rs3754274	0.238885
HGR0000083	NONdegraders-adult	DNASE2B	chr1	84867609	84867610	G	A	missense_v	MED	rs3754274	0.238885
HGR0000080	NONdegraders-adult	DNASE2B	chr1	84867609	84867610	G	A	missense_v	MED	rs3754274	0.238885
HGR0000065	NONdegraders-adult	DNASE2B	chr1	84867609	84867610	G	A	missense_v	MED	rs3754274	0.238885
HGR0000090	NONdegraders-adult	DNASE2B	chr1	84867609	84867610	G	A	missense_v	MED	rs3754274	0.238885
HGR0000092	NONdegraders-adult	DNASE2B	chr1	84867609	84867610	G	A	missense_v	MED	rs3754274	0.238885
HGR0000003	NONdegraders-adult	DNASE2B	chr1	84867609	84867610	G	A	missense_v	MED	rs3754274	0.238885
HGR0000074	NONdegraders-adult	DNASE2B	chr1	84867609	84867610	G	A	missense_v	MED	rs3754274	0.238885
HGR0000034	NONdegraders-adult	DNASE2B	chr1	84867609	84867610	G	A	missense_v	MED	rs3754274	0.238885
HGR0000081	NONdegraders-adult	DNASE2B	chr1	84867609	84867610	G	A	missense_v	MED	rs3754274	0.238885
HGR0000020	NONdegraders-adult	DNASE2B	chr1	84867609	84867610	G	A	missense_v	MED	rs3754274	0.238885
HGR0000086	NONdegraders-adult	DNASE2B	chr1	84867609	84867610	G	A	missense_v	MED	rs3754274	0.238885
HGR0000072	NONdegraders-adult	DNASE2B	chr1	84867609	84867610	G	A	missense_v	MED	rs3754274	0.238885
HGR0000017	NONdegraders-adult	DNASE2B	chr1	84867609	84867610	G	A	missense_v	MED	rs3754274	0.238885
HGR0000106	NONdegraders-adult	DNASE2B	chr1	84867609	84867610	G	A	missense_v	MED	rs3754274	0.238885
HGR0000088	NONdegraders-adult	DNASE2B	chr1	84867609	84867610	G	A	missense_v	MED	rs3754274	0.238885
HGR0000040	NONdegraders-adult	DNASE2B	chr1	84867609	84867610	G	A	missense_v	MED	rs3754274	0.238885
HGR0000033	NONdegraders-adult	DNASE2B	chr1	84867609	84867610	G	A	missense_v	MED	rs3754274	0.238885
HGR0000089	NONdegraders-adult	DNASE2B	chr1	84867609	84867610	G	A	missense_v	MED	rs3754274	0.238885
HGR0000044	NONdegraders-adult	DNASE2B	chr1	84867609	84867610	G	A	missense_v	MED	rs3754274	0.238885
HGR0000006	NONdegraders-adult	DNASE2B	chr1	84867609	84867610	G	A	missense_v	MED	rs3754274	0.238885
HGR0000037	NONdegraders-adult	DNASE2B	chr1	84867609	84867610	G	A	missense_v	MED	rs3754274	0.238885
HGR0000073	NONdegraders-adult	DNASE2B	chr1	84867609	84867610	G	A	missense_v	MED	rs3754274	0.238885
HGR0000063	NONdegraders-adult	DNASE2B	chr1	84867609	84867610	G	A	missense_v	MED	rs3754274	0.238885
HGR0000098	NONdegraders-adult	DNASE2B	chr1	84867609	84867610	G	A	missense_v	MED	rs3754274	0.238885
HGR0000019	NONdegraders-adult	DNASE2B	chr1	84867609	84867610	G	A	missense_v	MED	rs3754274	0.238885
HGR0000067	NONdegraders-adult	DNASE2B	chr1	84867609	84867610	G	A	missense_v	MED	rs3754274	0.238885
HGR0000085	NONdegraders-adult	DNASE2B	chr1	84867609	84867610	G	A	missense_v	MED	rs3754274	0.238885
HGR0001230	NONdegraders-adult	DNASE2B	chr1	84867609	84867610	G	A	missense_v	MED	rs3754274	0.238885
HGR0001227	NONdegraders-adult	DNASE2B	chr1	84867609	84867610	G	A	missense_v	MED	rs3754274	0.238885
HGR0001240	NONdegraders-adult	DNASE2B	chr1	84867609	84867610	G	A	missense_v	MED	rs3754274	0.238885
HGR0001225	Degraders-adult	DNASE1L3	chr3	58186718	58186719	C	A	splice_regio	MED	rs3772985	0.175386
HGR0001233	Degraders-adult	DNASE1L3	chr3	58186718	58186719	C	A	splice_regio	MED	rs3772985	0.175386
HGR0001226	Degraders-adult	DNASE1L3	chr3	58186718	58186719	C	A	splice_regio	MED	rs3772985	0.175386
HGR0000038	Degraders-adult	DNASE1L3	chr3	58186718	58186719	C	A	splice_regio	MED	rs3772985	0.175386
HGR0000110	Degraders-adult	DNASE1L3	chr3	58186718	58186719	C	A	splice_regio	MED	rs3772985	0.175386
HGR0000009	Degraders-adult	DNASE1L3	chr3	58186718	58186719	C	A	splice_regio	MED	rs3772985	0.175386
HGR0000018	Degraders-adult	DNASE1L3	chr3	58186718	58186719	C	A	splice_regio	MED	rs3772985	0.175386
HGR0000002	NONdegraders-adult	DNASE1L3	chr3	58186718	58186719	C	A	splice_regio	MED	rs3772985	0.175386
HGR0000098	NONdegraders-adult	DNASE1L3	chr3	58186718	58186719	C	A	splice_regio	MED	rs3772985	0.175386
HGR0000055	NONdegraders-adult	DNASE1L3	chr3	58186718	58186719	C	A	splice_regio	MED	rs3772985	0.175386
HGR0000091	NONdegraders-adult	DNASE1L3	chr3	58186718	58186719	C	A	splice_regio	MED	rs3772985	0.175386
HGR0000068	NONdegraders-adult	DNASE1L3	chr3	58186718	58186719	C	A	splice_regio	MED	rs3772985	0.175386
HGR0000010	NONdegraders-adult	DNASE1L3	chr3	58186718	58186719	C	A	splice_regio	MED	rs3772985	0.175386
HGR0000030	NONdegraders-adult	DNASE1L3	chr3	58186718	58186719	C	A	splice_regio	MED	rs3772985	0.175386
HGR0000079	NONdegraders-adult	DNASE1L3	chr3	58186718	58186719	C	A	splice_regio	MED	rs3772985	0.175386

HGR0001230	NONdegraders-adult	DNASE1L3	chr3	58186718	58186719	C	A	splice_regio	MED	rs3772985	0.175386
HGR0000075	NONdegraders-adult	DNASE1L3	chr3	58186718	58186719	C	A	splice_regio	MED	rs3772985	0.175386
HGR0000017	NONdegraders-adult	DNASE1L3	chr3	58186718	58186719	C	A	splice_regio	MED	rs3772985	0.175386
HGR0000046	NONdegraders-adult	DNASE1L3	chr3	58186718	58186719	C	A	splice_regio	MED	rs3772985	0.175386
HGR0001228	Degraders-adult	DNASE2B	chr1	84878227	84878228	C	T	splice_regio	MED	rs7511984	0.178255
HGR0001233	Degraders-adult	DNASE2B	chr1	84878227	84878228	C	T	splice_regio	MED	rs7511984	0.178255
HGR0000059	Degraders-adult	DNASE2B	chr1	84878227	84878228	C	T	splice_regio	MED	rs7511984	0.178255
HGR0000082	Degraders-adult	DNASE2B	chr1	84878227	84878228	C	T	splice_regio	MED	rs7511984	0.178255
HGR0001224	Degraders-adult	DNASE2B	chr1	84878227	84878228	C	T	splice_regio	MED	rs7511984	0.178255
HGR0000032	Degraders-adult	DNASE2B	chr1	84878227	84878228	C	T	splice_regio	MED	rs7511984	0.178255
HGR0001239	Degraders-adult	DNASE2B	chr1	84878227	84878228	C	T	splice_regio	MED	rs7511984	0.178255
HGR0001225	Degraders-adult	DNASE2B	chr1	84878227	84878228	C	T	splice_regio	MED	rs7511984	0.178255
HGR0000026	Degraders-adult	DNASE2B	chr1	84878227	84878228	C	T	splice_regio	MED	rs7511984	0.178255
HGR0000058	Degraders-adult	DNASE2B	chr1	84878227	84878228	C	T	splice_regio	MED	rs7511984	0.178255
HGR0000022	NONdegraders-adult	DNASE2B	chr1	84878227	84878228	C	T	splice_regio	MED	rs7511984	0.178255
HGR0000034	NONdegraders-adult	DNASE2B	chr1	84878227	84878228	C	T	splice_regio	MED	rs7511984	0.178255
HGR0000036	NONdegraders-adult	DNASE2B	chr1	84878227	84878228	C	T	splice_regio	MED	rs7511984	0.178255
HGR0000042	NONdegraders-adult	DNASE2B	chr1	84878227	84878228	C	T	splice_regio	MED	rs7511984	0.178255
HGR0000044	NONdegraders-adult	DNASE2B	chr1	84878227	84878228	C	T	splice_regio	MED	rs7511984	0.178255
HGR0001238	NONdegraders-adult	DNASE2B	chr1	84878227	84878228	C	T	splice_regio	MED	rs7511984	0.178255
HGR0000077	NONdegraders-adult	DNASE2B	chr1	84878227	84878228	C	T	splice_regio	MED	rs7511984	0.178255
HGR0000080	NONdegraders-adult	DNASE2B	chr1	84878227	84878228	C	T	splice_regio	MED	rs7511984	0.178255
HGR0000047	NONdegraders-adult	DNASE2B	chr1	84878227	84878228	C	T	splice_regio	MED	rs7511984	0.178255
HGR0000049	NONdegraders-adult	DNASE2B	chr1	84878227	84878228	C	T	splice_regio	MED	rs7511984	0.178255
HGR0000021	NONdegraders-adult	DNASE2B	chr1	84878227	84878228	C	T	splice_regio	MED	rs7511984	0.178255
HGR0000093	NONdegraders-adult	DNASE2B	chr1	84878227	84878228	C	T	splice_regio	MED	rs7511984	0.178255
HGR0000063	NONdegraders-adult	DNASE2B	chr1	84878227	84878228	C	T	splice_regio	MED	rs7511984	0.178255
HGR0000074	NONdegraders-adult	DNASE2B	chr1	84878227	84878228	C	T	splice_regio	MED	rs7511984	0.178255
HGR0000078	NONdegraders-adult	DNASE2B	chr1	84878227	84878228	C	T	splice_regio	MED	rs7511984	0.178255
HGR0000081	NONdegraders-adult	DNASE2B	chr1	84878227	84878228	C	T	splice_regio	MED	rs7511984	0.178255
HGR0001229	NONdegraders-adult	DNASE2B	chr1	84878227	84878228	C	T	splice_regio	MED	rs7511984	0.178255
HGR0000086	NONdegraders-adult	DNASE2B	chr1	84878227	84878228	C	T	splice_regio	MED	rs7511984	0.178255
HGR0000028	NONdegraders-adult	DNASE2B	chr1	84878227	84878228	C	T	splice_regio	MED	rs7511984	0.178255
HGR0000075	NONdegraders-adult	DNASE2B	chr1	84878227	84878228	C	T	splice_regio	MED	rs7511984	0.178255

HOMOZYGOUS RECESSIVE

HGRepoID	*Category*	*gene*	*chrom*	*start*	*end*	*ref*	*alt*	*impact*	*impact_sev*	*rs_ids*	*gnomad_MAF*
HGR0001232	Degraders-adult	DNASE1	chr16	3707746	3707747	G	A	missense_v	MED	rs1053874	0.444635
HGR0000079	NONdegraders-adult	DNASE1	chr16	3707746	3707747	G	A	missense_v	MED	rs1053874	0.444635
HGR0000003	NONdegraders-adult	DNASE1	chr16	3707746	3707747	G	A	missense_v	MED	rs1053874	0.444635
HGR0000069	NONdegraders-adult	DNASE1	chr16	3707746	3707747	G	A	missense_v	MED	rs1053874	0.444635
HGR0000071	NONdegraders-adult	DNASE1	chr16	3707746	3707747	G	A	missense_v	MED	rs1053874	0.444635
HGR0000090	NONdegraders-adult	DNASE1	chr16	3707746	3707747	G	A	missense_v	MED	rs1053874	0.444635
HGR0000022	NONdegraders-adult	DNASE1	chr16	3707746	3707747	G	A	missense_v	MED	rs1053874	0.444635
HGR0001235	Degraders-adult	DNASE2B	chr1	84864255	84864256	G	C	missense_v	MED	rs3738573	0.321401
HGR0001234	Degraders-adult	DNASE2B	chr1	84864255	84864256	G	C	missense_v	MED	rs3738573	0.321401
HGR0000107	Degraders-adult	DNASE2B	chr1	84864255	84864256	G	C	missense_v	MED	rs3738573	0.321401
HGR0000094	Degraders-adult	DNASE2B	chr1	84864255	84864256	G	C	missense_v	MED	rs3738573	0.321401
HGR0000111	Degraders-adult	DNASE2B	chr1	84864255	84864256	G	C	missense_v	MED	rs3738573	0.321401
HGR0000100	Degraders-adult	DNASE2B	chr1	84864255	84864256	G	C	missense_v	MED	rs3738573	0.321401
HGR0000001	Degraders-adult	DNASE2B	chr1	84864255	84864256	G	C	missense_v	MED	rs3738573	0.321401
HGR0000027	Degraders-adult	DNASE2B	chr1	84864255	84864256	G	C	missense_v	MED	rs3738573	0.321401
HGR0000060	Degraders-adult	DNASE2B	chr1	84864255	84864256	G	C	missense_v	MED	rs3738573	0.321401
HGR0000046	NONdegraders-adult	DNASE2B	chr1	84864255	84864256	G	C	missense_v	MED	rs3738573	0.321401
HGR0000057	NONdegraders-adult	DNASE2B	chr1	84864255	84864256	G	C	missense_v	MED	rs3738573	0.321401
HGR0000072	NONdegraders-adult	DNASE2B	chr1	84864255	84864256	G	C	missense_v	MED	rs3738573	0.321401
HGR0000051	NONdegraders-adult	DNASE2B	chr1	84864255	84864256	G	C	missense_v	MED	rs3738573	0.321401
HGR0000066	NONdegraders-adult	DNASE2B	chr1	84864255	84864256	G	C	missense_v	MED	rs3738573	0.321401
HGR0001235	Degraders-adult	DNASE2B	chr1	84867609	84867610	G	A	missense_v	MED	rs3754274	0.238885
HGR0000094	Degraders-adult	DNASE2B	chr1	84867609	84867610	G	A	missense_v	MED	rs3754274	0.238885
HGR0000100	Degraders-adult	DNASE2B	chr1	84867609	84867610	G	A	missense_v	MED	rs3754274	0.238885
HGR0000107	Degraders-adult	DNASE2B	chr1	84867609	84867610	G	A	missense_v	MED	rs3754274	0.238885
HGR0000027	Degraders-adult	DNASE2B	chr1	84867609	84867610	G	A	missense_v	MED	rs3754274	0.238885
HGR0000111	Degraders-adult	DNASE2B	chr1	84867609	84867610	G	A	missense_v	MED	rs3754274	0.238885
HGR0000068	NONdegraders-adult	DNASE2B	chr1	84867609	84867610	G	A	missense_v	MED	rs3754274	0.238885
HGR0000051	NONdegraders-adult	DNASE2B	chr1	84867609	84867610	G	A	missense_v	MED	rs3754274	0.238885
HGR0000066	NONdegraders-adult	DNASE2B	chr1	84867609	84867610	G	A	missense_v	MED	rs3754274	0.238885
HGR0000046	NONdegraders-adult	DNASE2B	chr1	84867609	84867610	G	A	missense_v	MED	rs3754274	0.238885
HGR0000050	NONdegraders-adult	DNASE2B	chr1	84867609	84867610	G	A	missense_v	MED	rs3754274	0.238885
HGR0000084	Degraders-adult	DNASE2B	chr1	84878227	84878228	C	T	splice_regio	MED	rs7511984	0.178255
HGR0000053	NONdegraders-adult	DNASE2B	chr1	84878227	84878228	C	T	splice_regio	MED	rs7511984	0.178255
HGR0000012	NONdegraders-adult	DNASE2B	chr1	84878227	84878228	C	T	splice_regio	MED	rs7511984	0.178255
HGR0000039	NONdegraders-adult	DNASE2B	chr1	84878227	84878228	C	T	splice_regio	MED	rs7511984	0.178255

COMPOUND HETERO											
HGRepoID	***Category***	***gene***	***chrom***	***start***	***end***	***ref***	***alt***	***impact***	***impact_sev***	***rs_ids***	***gnomad_MAF***
HGR0000110	Degraders-adult	DNASE1	chr16	3706667	3706668	A	C	missense_v	MED	rs34923865	0.0110644
HGR0000110	Degraders-adult	DNASE1	chr16	3707746	3707747	G	A	missense_v	MED	rs1053874	0.444635
HGR0001235	Degraders-adult	DNASE1	chr16	3705379	3705380	G	T	missense_v	MED	rs8176927	0.0340248
HGR0001235	Degraders-adult	DNASE1	chr16	3707746	3707747	G	A	missense_v	MED	rs1053874	0.444635
HGR0000038	Degraders-adult	DNASE1L3	chr3	58183635	58183636	G	A	missense_v	MED	rs35677470	0.0500614
HGR0000038	Degraders-adult	DNASE1L3	chr3	58186718	58186719	C	A	splice_regio	MED	rs3772985	0.175386
HGR0001226	Degraders-adult	DNASE1L3	chr3	58183635	58183636	G	A	missense_v	MED	rs35677470	0.0500614
HGR0001226	Degraders-adult	DNASE1L3	chr3	58186718	58186719	C	A	splice_regio	MED	rs3772985	0.175386
HGR0000009	Degraders-adult	DNASE2B	chr1	84864255	84864256	G	C	missense_v	MED	rs3738573	0.321401
HGR0000009	Degraders-adult	DNASE2B	chr1	84867609	84867610	G	A	missense_v	MED	rs3754274	0.238885
HGR0000013	Degraders-adult	DNASE2B	chr1	84864255	84864256	G	C	missense_v	MED	rs3738573	0.321401
HGR0000013	Degraders-adult	DNASE2B	chr1	84867609	84867610	G	A	missense_v	MED	rs3754274	0.238885
HGR0000014	Degraders-adult	DNASE2B	chr1	84864255	84864256	G	C	missense_v	MED	rs3738573	0.321401
HGR0000014	Degraders-adult	DNASE2B	chr1	84876679	84876680	T	C	missense_v	MED	rs149919241	0.00152056
HGR0000023	Degraders-adult	DNASE2B	chr1	84864255	84864256	G	C	missense_v	MED	rs3738573	0.321401
HGR0000023	Degraders-adult	DNASE2B	chr1	84867609	84867610	G	A	missense_v	MED	rs3754274	0.238885
HGR0000038	Degraders-adult	DNASE2B	chr1	84864255	84864256	G	C	missense_v	MED	rs3738573	0.321401
HGR0000038	Degraders-adult	DNASE2B	chr1	84867609	84867610	G	A	missense_v	MED	rs3754274	0.238885
HGR0000054	Degraders-adult	DNASE2B	chr1	84864255	84864256	G	C	missense_v	MED	rs3738573	0.321401
HGR0000054	Degraders-adult	DNASE2B	chr1	84867609	84867610	G	A	missense_v	MED	rs3754274	0.238885
HGR0000056	Degraders-adult	DNASE2B	chr1	84864255	84864256	G	C	missense_v	MED	rs3738573	0.321401
HGR0000056	Degraders-adult	DNASE2B	chr1	84867609	84867610	G	A	missense_v	MED	rs3754274	0.238885
HGR0000061	Degraders-adult	DNASE2B	chr1	84864255	84864256	G	C	missense_v	MED	rs3738573	0.321401
HGR0000061	Degraders-adult	DNASE2B	chr1	84867609	84867610	G	A	missense_v	MED	rs3754274	0.238885
HGR0000062	Degraders-adult	DNASE2B	chr1	84864255	84864256	G	C	missense_v	MED	rs3738573	0.321401
HGR0000062	Degraders-adult	DNASE2B	chr1	84867609	84867610	G	A	missense_v	MED	rs3754274	0.238885
HGR0000082	Degraders-adult	DNASE2B	chr1	84878227	84878228	C	T	splice_regio	MED	rs7511984	0.178255
HGR0000082	Degraders-adult	DNASE2B	chr1	84864255	84864256	G	C	missense_v	MED	rs3738573	0.321401
HGR0000082	Degraders-adult	DNASE2B	chr1	84867609	84867610	G	A	missense_v	MED	rs3754274	0.238885
HGR0000101	Degraders-adult	DNASE2B	chr1	84864255	84864256	G	C	missense_v	MED	rs3738573	0.321401
HGR0000101	Degraders-adult	DNASE2B	chr1	84867609	84867610	G	A	missense_v	MED	rs3754274	0.238885
HGR0001228	Degraders-adult	DNASE2B	chr1	84878227	84878228	C	T	splice_regio	MED	rs7511984	0.178255
HGR0001228	Degraders-adult	DNASE2B	chr1	84864255	84864256	G	C	missense_v	MED	rs3738573	0.321401
HGR0001228	Degraders-adult	DNASE2B	chr1	84867609	84867610	G	A	missense_v	MED	rs3754274	0.238885
HGR0001236	Degraders-adult	DNASE2B	chr1	84864255	84864256	G	C	missense_v	MED	rs3738573	0.321401
HGR0001236	Degraders-adult	DNASE2B	chr1	84876679	84876680	T	C	missense_v	MED	rs149919241	0.00152056
HGR0000003	NONdegraders-adult	DNASE2B	chr1	84864255	84864256	G	C	missense_v	MED	rs3738573	0.321401
HGR0000003	NONdegraders-adult	DNASE2B	chr1	84867609	84867610	G	A	missense_v	MED	rs3754274	0.238885
HGR0000006	NONdegraders-adult	DNASE2B	chr1	84864255	84864256	G	C	missense_v	MED	rs3738573	0.321401
HGR0000006	NONdegraders-adult	DNASE2B	chr1	84867609	84867610	G	A	missense_v	MED	rs3754274	0.238885
HGR0000017	NONdegraders-adult	DNASE2B	chr1	84864255	84864256	G	C	missense_v	MED	rs3738573	0.321401
HGR0000017	NONdegraders-adult	DNASE2B	chr1	84867609	84867610	G	A	missense_v	MED	rs3754274	0.238885
HGR0000019	NONdegraders-adult	DNASE2B	chr1	84864255	84864256	G	C	missense_v	MED	rs3738573	0.321401
HGR0000019	NONdegraders-adult	DNASE2B	chr1	84867609	84867610	G	A	missense_v	MED	rs3754274	0.238885
HGR0000020	NONdegraders-adult	DNASE2B	chr1	84864255	84864256	G	C	missense_v	MED	rs3738573	0.321401
HGR0000020	NONdegraders-adult	DNASE2B	chr1	84867609	84867610	G	A	missense_v	MED	rs3754274	0.238885
HGR0000033	NONdegraders-adult	DNASE2B	chr1	84864255	84864256	G	C	missense_v	MED	rs3738573	0.321401
HGR0000033	NONdegraders-adult	DNASE2B	chr1	84867609	84867610	G	A	missense_v	MED	rs3754274	0.238885
HGR0000034	NONdegraders-adult	DNASE2B	chr1	84878227	84878228	C	T	splice_regio	MED	rs7511984	0.178255
HGR0000034	NONdegraders-adult	DNASE2B	chr1	84864255	84864256	G	C	missense_v	MED	rs3738573	0.321401
HGR0000034	NONdegraders-adult	DNASE2B	chr1	84867609	84867610	G	A	missense_v	MED	rs3754274	0.238885
HGR0000036	NONdegraders-adult	DNASE2B	chr1	84878227	84878228	C	T	splice_regio	MED	rs7511984	0.178255
HGR0000036	NONdegraders-adult	DNASE2B	chr1	84864255	84864256	G	C	missense_v	MED	rs3738573	0.321401
HGR0000037	NONdegraders-adult	DNASE2B	chr1	84864255	84864256	G	C	missense_v	MED	rs3738573	0.321401
HGR0000037	NONdegraders-adult	DNASE2B	chr1	84867609	84867610	G	A	missense_v	MED	rs3754274	0.238885
HGR0000040	NONdegraders-adult	DNASE2B	chr1	84864255	84864256	G	C	missense_v	MED	rs3738573	0.321401
HGR0000040	NONdegraders-adult	DNASE2B	chr1	84867609	84867610	G	A	missense_v	MED	rs3754274	0.238885
HGR0000044	NONdegraders-adult	DNASE2B	chr1	84878227	84878228	C	T	splice_regio	MED	rs7511984	0.178255
HGR0000044	NONdegraders-adult	DNASE2B	chr1	84864255	84864256	G	C	missense_v	MED	rs3738573	0.321401
HGR0000044	NONdegraders-adult	DNASE2B	chr1	84867609	84867610	G	A	missense_v	MED	rs3754274	0.238885
HGR0000047	NONdegraders-adult	DNASE2B	chr1	84878227	84878228	C	T	splice_regio	MED	rs7511984	0.178255
HGR0000047	NONdegraders-adult	DNASE2B	chr1	84864255	84864256	G	C	missense_v	MED	rs3738573	0.321401
HGR0000063	NONdegraders-adult	DNASE2B	chr1	84878227	84878228	C	T	splice_regio	MED	rs7511984	0.178255
HGR0000063	NONdegraders-adult	DNASE2B	chr1	84864255	84864256	G	C	missense_v	MED	rs3738573	0.321401
HGR0000063	NONdegraders-adult	DNASE2B	chr1	84867609	84867610	G	A	missense_v	MED	rs3754274	0.238885
HGR0000065	NONdegraders-adult	DNASE2B	chr1	84864255	84864256	G	C	missense_v	MED	rs3738573	0.321401
HGR0000065	NONdegraders-adult	DNASE2B	chr1	84867609	84867610	G	A	missense_v	MED	rs3754274	0.238885
HGR0000067	NONdegraders-adult	DNASE2B	chr1	84864255	84864256	G	C	missense_v	MED	rs3738573	0.321401
HGR0000067	NONdegraders-adult	DNASE2B	chr1	84867609	84867610	G	A	missense_v	MED	rs3754274	0.238885

HGR0000073	NONdegraders-adult	DNASE2B	chr1	84864255	84864256	G	C	missense_v	MED	rs3738573	0.321401
HGR0000073	NONdegraders-adult	DNASE2B	chr1	84867609	84867610	G	A	missense_v	MED	rs3754274	0.238885
HGR0000074	NONdegraders-adult	DNASE2B	chr1	84878227	84878228	C	T	splice_regio	MED	rs7511984	0.178255
HGR0000074	NONdegraders-adult	DNASE2B	chr1	84864255	84864256	G	C	missense_v	MED	rs3738573	0.321401
HGR0000074	NONdegraders-adult	DNASE2B	chr1	84867609	84867610	G	A	missense_v	MED	rs3754274	0.238885
HGR0000075	NONdegraders-adult	DNASE2B	chr1	84878227	84878228	C	T	splice_regio	MED	rs7511984	0.178255
HGR0000075	NONdegraders-adult	DNASE2B	chr1	84864255	84864256	G	C	missense_v	MED	rs3738573	0.321401
HGR0000077	NONdegraders-adult	DNASE2B	chr1	84878227	84878228	C	T	splice_regic	MED	rs7511984	0.178255
HGR0000077	NONdegraders-adult	DNASE2B	chr1	84864255	84864256	G	C	missense_v	MED	rs3738573	0.321401
HGR0000078	NONdegraders-adult	DNASE2B	chr1	84878227	84878228	C	T	splice_regic	MED	rs7511984	0.178255
HGR0000078	NONdegraders-adult	DNASE2B	chr1	84864255	84864256	G	C	missense_v	MED	rs3738573	0.321401
HGR0000080	NONdegraders-adult	DNASE2B	chr1	84878227	84878228	C	T	splice_regic	MED	rs7511984	0.178255
HGR0000080	NONdegraders-adult	DNASE2B	chr1	84864255	84864256	G	C	missense_v	MED	rs3738573	0.321401
HGR0000080	NONdegraders-adult	DNASE2B	chr1	84867609	84867610	G	A	missense_v	MED	rs3754274	0.238885
HGR0000081	NONdegraders-adult	DNASE2B	chr1	84878227	84878228	C	T	splice_regic	MED	rs7511984	0.178255
HGR0000081	NONdegraders-adult	DNASE2B	chr1	84864255	84864256	G	C	missense_v	MED	rs3738573	0.321401
HGR0000081	NONdegraders-adult	DNASE2B	chr1	84867609	84867610	G	A	missense_v	MED	rs3754274	0.238885
HGR0000083	NONdegraders-adult	DNASE2B	chr1	84864255	84864256	G	C	missense_v	MED	rs3738573	0.321401
HGR0000083	NONdegraders-adult	DNASE2B	chr1	84867609	84867610	G	A	missense_v	MED	rs3754274	0.238885
HGR0000085	NONdegraders-adult	DNASE2B	chr1	84864255	84864256	G	C	missense_v	MED	rs3738573	0.321401
HGR0000085	NONdegraders-adult	DNASE2B	chr1	84867609	84867610	G	A	missense_v	MED	rs3754274	0.238885
HGR0000086	NONdegraders-adult	DNASE2B	chr1	84878227	84878228	C	T	splice_regic	MED	rs7511984	0.178255
HGR0000086	NONdegraders-adult	DNASE2B	chr1	84864255	84864256	G	C	missense_v	MED	rs3738573	0.321401
HGR0000086	NONdegraders-adult	DNASE2B	chr1	84867609	84867610	G	A	missense_v	MED	rs3754274	0.238885
HGR0000088	NONdegraders-adult	DNASE2B	chr1	84864255	84864256	G	C	missense_v	MED	rs3738573	0.321401
HGR0000088	NONdegraders-adult	DNASE2B	chr1	84867609	84867610	G	A	missense_v	MED	rs3754274	0.238885
HGR0000089	NONdegraders-adult	DNASE2B	chr1	84864255	84864256	G	C	missense_v	MED	rs3738573	0.321401
HGR0000089	NONdegraders-adult	DNASE2B	chr1	84867609	84867610	G	A	missense_v	MED	rs3754274	0.238885
HGR0000090	NONdegraders-adult	DNASE2B	chr1	84864255	84864256	G	C	missense_v	MED	rs3738573	0.321401
HGR0000090	NONdegraders-adult	DNASE2B	chr1	84867609	84867610	G	A	missense_v	MED	rs3754274	0.238885
HGR0000092	NONdegraders-adult	DNASE2B	chr1	84864255	84864256	G	C	missense_v	MED	rs3738573	0.321401
HGR0000092	NONdegraders-adult	DNASE2B	chr1	84867609	84867610	G	A	missense_v	MED	rs3754274	0.238885
HGR0000098	NONdegraders-adult	DNASE2B	chr1	84864255	84864256	G	C	missense_v	MED	rs3738573	0.321401
HGR0000098	NONdegraders-adult	DNASE2B	chr1	84867609	84867610	G	A	missense_v	MED	rs3754274	0.238885
HGR0000106	NONdegraders-adult	DNASE2B	chr1	84864255	84864256	G	C	missense_v	MED	rs3738573	0.321401
HGR0000106	NONdegraders-adult	DNASE2B	chr1	84867609	84867610	G	A	missense_v	MED	rs3754274	0.238885
HGR0001227	NONdegraders-adult	DNASE2B	chr1	84864255	84864256	G	C	missense_v	MED	rs3738573	0.321401
HGR0001227	NONdegraders-adult	DNASE2B	chr1	84867609	84867610	G	A	missense_v	MED	rs3754274	0.238885
HGR0001230	NONdegraders-adult	DNASE2B	chr1	84864255	84864256	G	C	missense_v	MED	rs3738573	0.321401
HGR0001230	NONdegraders-adult	DNASE2B	chr1	84867609	84867610	G	A	missense_v	MED	rs3754274	0.238885
HGR0001240	NONdegraders-adult	DNASE2B	chr1	84864255	84864256	G	C	missense_v	MED	rs3738573	0.321401
HGR0001240	NONdegraders-adult	DNASE2B	chr1	84867609	84867610	G	A	missense_v	MED	rs3754274	0.238885

Supplementary Table 5. Summary of detection of NET remnants and degradation per cohort studied

Cohort	NETs	Degradation
MIS-C (Italy)	no change	Impaired
MIS-C (Chile)	elevated	Impaired
CLL (Italy)	elevated	no change
CLL (USA)	elevated	Impaired
Covid19 symptomatic	elevated	Impaired
Covid19 asymptomatic	no change	no change
Covid19 (Omicron)	no change	

Supplementary Table 6. Demographic characteristics of healthy controls and patients used for each Figure

Figure 1-3

**Pediatric control (n=19)**		**Italian MIS-C cohort (n=6)**		**Italian CLL cohort (n=17)**		**Chile MIS-C cohort (n=27)**		**USA CLL cohort (n=9)**	
Age: 4 (1.8-6)		Age: 5.8 (0.3-12)		Age: 13 (9-15)		Age: 6 (3-11)		Age: 17 (14-19.5)	
**Feature**	**Number (%)**	**Feature**	**Number (%)**	**Feature**	**Number (%)**	**Feature**	**Number (%)**	**Feature**	**Number (%)**
Male	N/A	Male	3 (50)	Male	9 (52.9)	Male	16 (59)	Male	5 (55.5)

Figure 4-6

**Adult Control (n=13)**		**Adult cohorts (n=280)**	
Age: 56 (35- 63.5)		Age: 60 (19-92)	
**Feature**	**Number (%)**	**Feature**	**Number (%)**
Male	5 (38)	Male	192 (68.6)

Figure-8

**Control (n=14)**		**Alpha (n=14)**		**Omicron (n=21)**	
Age: 54 (40.5- 57)		Age: 60.5 (48-69)		Age:73 (64-85)	
**Feature**	**Number (%)**	**Feature**	**Number (%)**	**Feature**	**Number (%)**
Male	2 (14)	Male	12(86)	Male	13 (62)

Supplementary Table 7. Patients that underwent WGS .

rsID	Gnomad MAD	Conflict degrader status - MISC (n=7)	Degraders-MISC (n=17)	NONdegraders-MISC (n=48)	Degraders-adult (n=49)	NONdegraders-adult (n=81)
rs1053874	0.444635	0.428571	0.176471	0.208333	0.234694	0.129630
rs3738573	0.321401	0.142857	0.352941	0.250000	0.204082	0.364198
rs3754274	0.238885	0.357143	0.294118	0.239583	0.265306	0.246914
rs7511984	0.178255	0.142857	0.264706	0.062500	0.122449	0.160494
rs3772985	0.175386	0.071429	0.205882	0.114583	0.071429	0.074074
rs160550	0.088690	0.000000	0.000000	0.031250	0.000000	0.006173
rs35677470	0.050061	0.000000	0.029412	0.020833	0.040816	0.006173
rs8176927	0.034025	0.000000	0.000000	0.010417	0.010204	0.006173
rs34952165	0.033192	0.071429	0.029412	0.010417	0.030612	0.030864
rs76042396	0.031510	0.000000	0.000000	0.010417	0.000000	0.000000
rs150564609,rs397957651	0.015731	0.000000	0.000000	0.010417	0.000000	0.000000
rs34923865	0.011064	0.000000	0.029412	0.000000	0.010204	0.006173
rs74350392	0.009006	0.000000	0.000000	0.000000	0.000000	0.030864
rs12491947	0.006717	0.000000	0.029412	0.052083	0.000000	0.000000
rs191517595	0.005206	0.000000	0.029412	0.000000	0.000000	0.000000
rs112348773	0.003716	0.000000	0.000000	0.000000	0.000000	0.006173
rs34907394	0.003490	0.071429	0.000000	0.000000	0.000000	0.018519
rs62621282	0.003239	0.000000	0.000000	0.000000	0.010204	0.006173
rs1799891	0.002427	0.000000	0.000000	0.041667	0.000000	0.000000
rs200659060	0.002166	0.000000	0.000000	0.000000	0.010204	0.006173
rs149919241	0.001521	0.000000	0.000000	0.000000	0.020408	0.000000
rs148373909	0.001099	0.000000	0.000000	0.020833	0.000000	0.000000
rs149357200	0.000744	0.000000	0.000000	0.000000	0.000000	0.006173

rs200934792	0.000422	0.000000	0.000000	0.000000	0.010204	0.000000
rs144754107	0.000234	0.000000	0.000000	0.010417	0.000000	0.000000
rs141205509	0.000234	0.000000	0.000000	0.010417	0.000000	0.000000
rs143058517	0.000194	0.000000	0.000000	0.010417	0.000000	0.000000
rs766377327	0.000129	0.000000	0.000000	0.000000	0.010204	0.000000
rs751822160	0.000099	0.000000	0.000000	0.000000	0.000000	0.006173
rs142482733	0.000032	0.071429	0.000000	0.000000	0.000000	0.006173
rs200773268	0.000032	0.000000	0.029412	0.000000	0.000000	0.000000
rs758521781	0.000000	0.071429	0.000000	0.000000	0.000000	0.000000
rs948201418	0.000000	0.071429	0.000000	0.000000	0.000000	0.000000
rs772354278	0.000000	0.000000	0.000000	0.000000	0.010204	0.000000
rs759268606	0.000000	0.000000	0.000000	0.000000	0.000000	0.006173
rs747122870	0.000000	0.000000	0.000000	0.000000	0.000000	0.006173

Supplementary Table 8. Test of reproducibility of serum/plasma samples after freeze and thaw cycles or heating at 60C .

	cit-H3-DNA/ Ela-DNA					cit-H3-DNA/ Ela-DNA	
**Plasma**	**first round**		**2nd round**			**non heated**	**heated at 60C for 30 min**
Sample-1	4.68		2.09		sample-1	0.94	0.97
Sample-2	2.22		4.62		sample-2	0.98	0.97
Sample-3	3.67		5.20		sample-3	1.04	1.01
Sample-4	2.89		2.09		sample-4	1.08	1.05
Sample-5	1.66		1.93		sample-5	0.96	1.00
Sample-6	1.13		1.42		sample-6	7.46	5.87
Sample-7	6.54		3.51		sample-7	1.02	1.03
Sample-8	7.96		10.51		sample-8	1.64	1.14
Sample-9	1.52		3.66		sample-9	4.17	3.89
**Serum**					sample-10	1.02	1.02
Sample-10	1.10		1.13		sample-11	1.27	1.13
Sample-11	5.80		4.30		sample-12	1.01	0.93
Sample-12	0.98		1.13		sample-13	0.88	0.97
Sample-13	2.40		4.26		sample-14	0.91	0.93
Sample-14	2.15		1.60		sample-15	0.92	1.04
Sample-15	2.23		1.73		sample-16	2.31	3.28
Sample-16	1.80		0.81		sample-17	1.12	1.48
Sample-17	2.13		3.06		sample-18	0.69	0.76
Sample-18	0.70		0.89		sample-19	1.76	1.94
Sample-19	1.62		3.37		sample-20	0.78	0.60
Sample-20	1.11		1.03				
Sample-21	1.32		1.97				
Sample-22	1.80		2.11				
Sample-23	0.25		0.29				
Sample-24	0.82		1.10				
Sample-25	0.57		0.52				
Sample-26	1.07		1.97				
Sample-27	2.66		2.17				
